# Privacy-by-Design Approach to Generate Two Virtual Clinical Trials for Multiple Sclerosis and Release Them as Open Datasets: Evaluation Study

**DOI:** 10.2196/71297

**Published:** 2025-10-01

**Authors:** Stanislas Demuth, Olivia Rousseau, Igor Faddeenkov, Julien Paris, Jérôme De Sèze, Béatrice Baciotti, Marianne Payet, Morgan Guillaudeux, Alban-Félix Barreteau, David Laplaud, Gilles Edan, Pierre-Antoine Gourraud

**Affiliations:** 1 Center for Research in Transplantation and Translational Immunology, Institut national de la santé et de la recherche médicale (INSERM), Nantes Université, 30 boulevard Jean Monnet, Nantes, 44093, France, 33 (0) 240087410; 2 Institut national de la santé et de la recherche médicale (INSERM) 1434, Clinical investigation center, University Hospital of Strasbourg, Strasbourg, France; 3 Department of Neurology, University Hospital of Strasbourg, Strasbourg, France; 4 Biogen France S.A.S, Paris, France; 5 Neurology, Merck Santé S.A.S., an affiliate of Merck KGaA, Lyon, France; 6 Octopize, Mimethik Data, Nantes, France; 7 Department of Neurology, University Hospital of Nantes, Nantes, France; 8 Department of Neurology, University Hospital of Rennes, Rennes, France; 9 Data Clinic, Department of Public Health, University Hospital of Nantes, Nantes, France

**Keywords:** synthetic data, privacy, multiple sclerosis, anonymization, randomized clinical trial

## Abstract

**Background:**

Sharing information derived from individual patient data is restricted by regulatory frameworks due to privacy concerns. Generative artificial intelligence can generate shareable virtual patient populations as proxies for sensitive reference datasets. Explicit demonstration of privacy is demanded.

**Objective:**

This study evaluated whether a privacy-by-design technique called “avatars” can generate synthetic datasets replicating all reported information from randomized clinical trials (RCTs).

**Methods:**

We generated 2160 synthetic datasets from two phase 3 RCTs for patients with multiple sclerosis (NCT00213135 and NCT00906399; n=865 and 1516 patients) with different configurations to select one synthetic dataset with optimal privacy and utility for each. Several privacy metrics were computed, including protection against distance-based membership inference attacks. We assessed fidelity by comparing variable distributions and assessed utility by checking that all end points reported in the publications had the same effect directions, were within the reported 95% CIs, and had the same statistical significance.

**Results:**

Protection against membership inference attacks was the hardest privacy metric to optimize, but the technique yielded robust privacy and replication of the primary end points (in 72.5% and 80.8% of the 1080 generated datasets). Utility was uneven across the variables and end points, such that information about some end points could not be captured. With optimized generation configurations, we selected one dataset from each RCT replicating all efficacy end points of the placebo and approved treatment arms while maintaining satisfactory privacy (hidden rate: 85.0% and 93.2%).

**Conclusions:**

Generating synthetic RCT datasets replicating primary and secondary efficacy end points is possible while achieving a satisfactory and explicit level of privacy. To show the potential of this method to unlock health data sharing, we released both placebo arms as open datasets.

## Introduction

### Background

Medical practices are becoming increasingly data-driven, as empirical evidence is sought to inform all clinical decisions. While studies analyzing real-world data from electronic health records provide real-world evidence [1], randomized clinical trial (RCT) data provide the highest level of evidence to guide medical practices, as this methodology approaches experimental settings. Within the standard clinical development pipeline of drugs, phase 3 RCTs are the largest-scale and most critical studies. Their primary end points provide regulatory evidence to approve new treatments on the market, while secondary end points and post hoc subgroup analyses, although not conclusive, provide high-quality information to generate hypotheses [2]. RCT data are classically accessible through credentials on data-sharing platforms (eg, Vivli.org [3], ClinicalStudyDataRequest.com [4]) and analyzed in closed virtual work environments. Their accessibility is conditioned on a predefined analysis plan, which must be designed blindly. Individual patient data (IPD) from RCTs can be used for feasibility studies, estimating sample sizes necessary for RCTs, indirect treatment comparisons [5,6], as learning datasets for predictive model development, or as external control arms for clinical trials [7]. As such, sharing RCT data as open datasets has been advocated by European regulators [8], but the technical implementation standards for such policies are currently lacking.

The use and sharing of health data for clinical research are restricted by regulatory frameworks due to privacy concerns (eg, the General Data Protection Regulation in Europe and the Health Insurance Portability and Accountability Act in the United States). Privacy is commonly addressed by enforcing the usage control through credentialed access and data deidentifying (ie, removing direct identifiers), yielding pseudonymous datasets. However, this does not prevent indirect reidentification by unique combinations of variables [9,10]. For the French data protection board (Commission Nationale de l’informatique et des libertés; CNIL), truly anonymous data must demonstrate the impossibility of linkage to the originating person [11]. As conceptual guidance, 3 anonymization criteria have been postulated by the European Data Protection Board [9] and integrated into the General Data Protection Regulation: (1) singling out (ie, unique identity disclosure), which is the capacity to reidentify a person from the uniqueness of records in a dataset; (2) linkability, which is the ability to link records concerning the same person across different databases; and (3) inference (ie, sensitive attribute disclosure), which is the possibility to deduce sensitive information about a person from the dataset.

Synthetic data are computationally generated individual observations created using a purpose-built mathematical model or algorithm [12]. Their most disseminated use case is digital content creation (images or text) using generative artificial intelligence models such as generative adversarial networks (GANs) [13] or large language models [14]. In medicine, model-based generators typically rely on GANs or variational autoencoder architectures and are commonly used for data augmentation or privacy enhancement [15]. The utility of synthetic datasets may be assessed using fidelity (ie, similarity) metrics and generator robustness [16]. More specifically, analytical utility stems from the veracity of the information, assessed by replicating aggregated results. The model footprint reflects the complexity of the model [17]. As synthetic datasets are computer-generated rather than collected from real persons, they are assumed to be anonymous by design. Thus, they appear as an alternative to share the information of sensitive datasets by representing it as a set of virtual patients instead of as the mathematical formula of a predictive model. However, there is concern about privacy leakage due to the individual granularity of synthetic datasets [18-20]. Hence, there is a growing demand to explicitly assess privacy using quantitative metrics [11,21].

### State of the Art

The field of virtual RCTs originally aimed to simulate the effect of new treatments with individual-level modeling [22]. This requires biomechanical models and has been achieved, for instance, in radiology in cross-sectional settings by the Virtual Imaging Clinical Trials for Regulatory Evaluation, which tested 2 mammography modalities on simulated images through a physics-based model of x-ray transmission and simulated breast cancer lesions [23]. An agent-based simulation of the immune system activity with the multiple sclerosis (MS) TreatSim approach has been proposed in MS and could replicate the primary end point of the AFFIRM trial [24]. Yet, drug development with biomechanistic modeling has not been achieved at the level of the whole organism.

The generation of virtual RCTs through statistical modeling aims to capture the information of reference datasets at the population level and then use the model generatively to yield synthetic IPD replicating the statistical behavior of reference IPD. The proposed use cases include providing technical stakeholders with mock data generated from metadata to explore standard data models such as CDISC (Clinical Data Interchange Standards Consortium) [25]. Other works propose privacy enhancement for data sharing [26], data augmentation to overcome insufficient patient accrual [27], or “synthetic control arms” [28]. However, this last term has mostly been used so far to designate external control arms of matched IPD from real-world data, which is closer to the field of clinical trials emulation than computer-generated data [29,30].

Synthetic data generators typically take one modality of raw data (eg, images) or a single-table tabular dataset as a reference [31]. Yet, health datasets have more complex data structures [32]. Electronic health record–derived data are longitudinal with a document data model, requiring time-series models [33]. Graph autoencoders could generate multitable datasets by modeling patient trajectories as directed acyclic graphs [34]. The standard follow-up of RCTs eases the representation of IPD as vectors to use classical statistical models. GANs adapted to tabular data or feature-based machine learning models have been used, with decision trees yielding the best performance [28,35]. Previous works focused primarily on oncology and assessed the utility by replicability of the primary end point and did not assess privacy [26,28].

The replicability of the reference RCT may be assessed through the fidelity of the data point distributions and the analytical utility as the replication of the study results: estimate agreement, CI overlap, decision agreement, or standardized difference [26]. The preservation of some predictive capacity is generally part of the utility assessment of synthetic data generated from real-world data [36]. The generators developed from real-world data so far have claimed privacy through some privacy assessment at the model evaluation step. This assessment may rely on the risk of membership disclosure [21] or the accuracy of an adversarial algorithm to discriminate real from synthetic data [17]. The previous works generating synthetic RCT data have not assessed privacy.

A synthetic data generator called the “avatars” technique has recently been reported with a privacy-by-design approach [37]. Unlike generative artificial intelligence models, it has been primarily designed as an anonymization technique with explicit privacy assessment. The initial report showed that synthetic datasets could be generated with high privacy metrics while outperforming Conditional Transformation-Generative Adversarial Network (CT-GAN) [38] and Synthpop [39] in replicating the primary end point analyses of an RCT and a cohort study. However, to become effective proxies of sensitive IPD, synthetic data must demonstrate a wider utility than merely replication of the main analysis of a reference dataset.

### Objective

In this study, we generated 2 synthetic RCT datasets in MS from the CLARITY and ADVANCE phase 3 trials using the avatars technique. MS is the most frequent chronic autoimmune disease of the central nervous system, progressively impairing multiple neurological functions. The main course is marked by relapsing episodes of disabling symptoms, associated with the accumulation of demyelinating lesions assessed by T2-weighted magnetic resonance imaging (MRI) and gadolinium enhancement. The classical efficacy end points of RCTs evaluating disease-modifying treatments are the annualized relapse rate (ARR), rate of T2 and gadolinium-enhancing (GdE) lesions, and confirmed disability worsening (CDW). The 3- or 6-month confirmation of the latter aims to rule out reversible relapse-associated symptoms. MS activity can be decreased by treatments commonly referred to as “disease-modifying treatments.”

Here, we determined to what extent this privacy-by-design technique can generate anonymous virtual patient datasets that capture most of the information reported in RCT publications, including primary and secondary efficacy end points, as well as safety. This work enabled the release of the placebo arms of both synthetic datasets as open data with approval of the relevant stakeholders, thus demonstrating the potential of synthetic data for information sharing in medicine.

## Methods

### Reference Datasets

**Figure 1.**
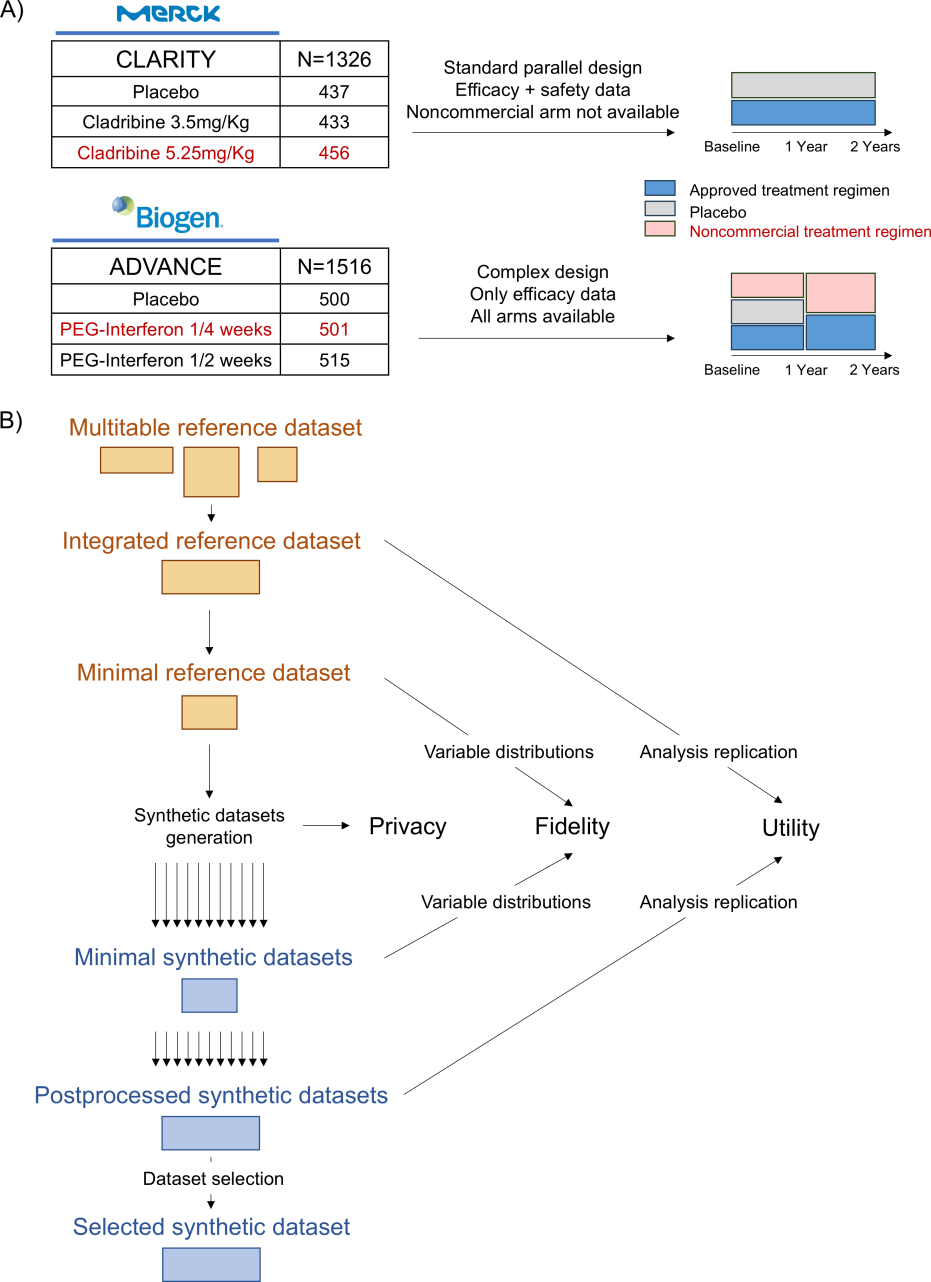
Reference datasets and pipeline of synthetic dataset generation and assessment. (A) Reference datasets were partially transferred as multiple tables and (B) the pipeline integrated the reference data into a single analysis-ready table for each RCT. To respect constraints between some variables, those with deterministic relations were removed, yielding minimal datasets for synthetic data generation. Several synthetic datasets were then generated with various parameter configurations. For every generated dataset, fidelity was assessed by comparing the minimal versions of reference and synthetic datasets, and utility was assessed by replicating the RCT analysis on the postprocessed versions. One dataset per RCT was selected based on the best privacy-utility trade-off, as described in the main text. PEG: pegylated; RCT: randomized clinical trial.

We used two independent phase 3 RCTs in MS as reference datasets: CLARITY from Merck (NCT00213135) [40] and ADVANCE from Biogen (NCT00906399) [41] (Figure 1). These trials were large-scale international studies whose primary end points provided regulatory evidence to approve 2 disease-modifying treatments for MS on the market: cladribine and peginterferon beta (Peg-IFNβ), respectively. CLARITY enrolled 1326 patients to test 2 regimens of cladribine versus placebo, and ADVANCE enrolled 1516 patients to test 2 regimens of Peg-IFNβ versus placebo. Both studies included patients without disease-modifying treatment for at least 3 months and lasted 2 years. The data were transferred after privacy-enhancement processes by both companies. For each RCT, we integrated the data into a single analysis-ready table (Figure 1). The variables were selected to replicate the graphical elements reported in the publications (ie, tables and flowcharts) as much as the transferred data enabled us to do. The primary efficacy end points that yielded the overall conclusion of the studies were the relapse activity. The secondary efficacy end points were the T2 and GdE MRI activity and CDW. Efficacy and safety data regarding adverse events (AEs) were available for 2 of the 3 arms of CLARITY: the placebo and the approved regimen (865 patients). We used CLARITY to assess whether synthetic datasets could capture the information on both efficacy and safety end points in the case of a classical parallel 2-arm design. We used ADVANCE to test the robustness of the technique for more complex study designs because the 3 arms were available, and patients in the placebo arm were rerandomized after 1 year to one of the 2 Peg-IFNβ regimens for the second year. However, only efficacy data were available.

### Synthetic Data Generation

The avatars technique was described in detail in its initial report [37]. Briefly, it generates synthetic data points using a multidimensional reduction and nearest neighbors algorithm. For each reference data point, the algorithm creates a local probability density model based on the topography of the nearest neighbors in the latent space of a factor analysis of mixed data (FAMD). A synthetic data point, called an “avatar,” is randomly sampled from the local model. In addition to standard privacy metrics, the 1:1 linkage of each avatar with its reference data point enables the assessment of the protection against membership inference attacks. The technique is proprietary and implemented in a client-server architecture (Octopize Mimetik). To help the technique respect the constraints between variables, we discarded variables with deterministic relations from the integrated analysis-ready table (eg, sum of 2 variables; Figure 1). The minimal dataset of CLARITY had 864 individual observations and 35 variables (7 categorical and 28 quantitative; Table S1 in Multimedia Appendix 1), and the minimal dataset of ADVANCE had 1512 individual observations and 25 variables (8 categorical and 17 quantitative; Table S2 in Multimedia Appendix 1). Each observation yields a reference data point. For quantitative variables, missing values were handled by default as “missing at random.” For categorical variables, we handled them as “missing not at random,” because they were related to study design and patient disposition. The Avatars server automatically imputes missing values with a k-nearest neighbors algorithm. We used the Python (version 0.7.2; Python Software Foundation) client of the avatars. Analogous to hyperparameter tuning in predictive model development, we tested different values of the following parameters to identify the configuration yielding the best compromise between privacy and utility:

1. k: the number of neighbors to create the local probabilistic model,
2. ncp: the number of projection components to compute the Euclidean distances of the neighbors, and
3. variable weights to favor a subset of variables during multidimensional reduction.

The tested values for k were 2, 5, 10, 15, 20, 25, 30, 40, 50, 75, 100, and 150, and the values tested for ncp were 5, 10, 20, 30, 46, and the maximum possible value. In our use case, ncp could be set up to 61 for CLARITY and up to 65 for ADVANCE, which is higher than the number of variables in the minimal datasets since categorical variables are automatically one-hot encoded by the Avatars server. The weighting of the variables was explored by preliminary generations to identify 2 relevant configurations per RCT in addition to the unweighted configuration. Alternative encodings of some variables were also tested, such as the encoding of relapse counts as categories (0, 1, 2, and 3 or more) and AEs count as Booleans (none vs any), and the handling of missing quantitative values as aberrant negative values instead of leaving them to be imputed by the Avatars server. Five synthetic datasets per configuration were generated with different random states for sampling avatars from the local probability density models. We used this random state as another hyperparameter. All generated datasets were analyzed separately (ie, no pooling). Finally, we removed patient identifiers and shuffled the rows of the selected synthetic datasets before release.

### Fidelity Assessment

Fidelity assessed the similarity of the synthetic dataset to the reference dataset regardless of its intended use: the similarity of univariate, bivariate, and multivariate distributions. All analyses were performed in R (version 4.2.3; The R Foundation). For univariate distributions, the Avatars server returned the mean of the Hellinger distances at the dataset level. Bivariate distributions of numeric variables were analyzed with the matrices of Pearson correlation coefficients returned by the Avatars server. Multivariate distributions were compared based on unweighted FAMD maps using the FactoMineR package (version 2.9 [42]) after multiple imputations with the MICE package (version 3.16.0 [43]). Weighted FAMD maps were also returned by the Avatars server using a dedicated Python algorithm developed by the software editor called SAIPH (Octopize Mimethik [44]).

### Utility Assessment

In this study, the utility assessed the similarity of the results obtained when replicating the analysis of interest on the synthetic dataset compared to those reported in the publications. For CLARITY, we also tested the replication of some post hoc subgroup analyses that proved critical for the market approval of cladribine [45]. We used R base functions, MASS [46], and the Survival packages (versions 7.3‐60 and 3.5‐7; Terry M Therneau) to replicate the statistical analysis based on the reported methods in the publications. For all end points, we considered the analysis to be replicated if (1) the estimate inferred from the synthetic dataset was within the 95% CIs reported in the publication, (2) the direction of the statistical effect was the same, and (3) the conclusion of the statistical test was the same (ie, whether the significance of the *P* value was <.05 or not). We estimated the 95% CIs of adjusted ARRs by nonparametric bootstrap with 1000 replications, using the Boot package (version 1.3‐28.1 [47]).

### Predictive Capacity

We also assessed the utility of the synthetic datasets for an alternative downstream task: the binary classification of patients who will have some MS activity during the study or not, either as clinical relapses or new MRI lesions. This predictive analysis included only complete cases. For ADVANCE, we have 1-year end points as targets because of the rerandomization of treatments for the placebo arm. Using the scikit-learn Python library (version 1.6.1 [48]), we trained and evaluated a random forest binary classifier for each end point (train-test split of 70%‐30%). We designated the “reference model” as the one developed on the reference dataset and the “test model” as the one developed on synthetic datasets (default or optimized configurations). We assessed the predictive performance through the area under the ROC curve and the accuracy. Their 95% CIs were estimated through bootstrapping (1000 resamplings). We also evaluated the generalizability of the performances of the reference and test models on the other dataset version, namely the synthetic and reference datasets, respectively.

### Privacy Assessment

Privacy was assessed by the privacy metrics returned by the Avatars server. They are defined briefly in Table 1, [49], and in detail on Octopize’s website [49]. The hidden rate (HR) is specific to the avatars technique and measures the risk of membership inference attacks [21]. It is computed from the local cloaking (LC) whose development has been detailed in the report of the avatar technique [37]. Briefly, for each patient, the LC counts the number of avatars that are more similar to his or her reference data point than his or her own avatar. An LC ≥1 means that a distance-based matching would be erroneous for this patient. This scenario is extreme because the attacker should know all the variables of the patient. In our case, the scenario would be that an attacker with access to the synthetic dataset attempts to assess whether the patient was enrolled in the RCT and thus infer his or her diagnosis of MS. At the dataset level, privacy is summarized by the median LC and the HR, which is the proportion of patients with an LC of ≥1. The software editor provides indicative targets for each metric (Table 1). In this study, we considered a median LC of 2 and an HR above 80% to be satisfactory.

**Table 1.** Privacy metrics of the selected datasets generated with optimized parameters. Metrics are grouped according to the conceptual anonymization criteria postulated by the European Data Protection Board. Detailed metric definitions are available on the software editor’s website. All distances are Euclidean.

Anonymization criteria and metric	Definition	Software editor recommendation (indicative)	CLARITY (optimized parameter)	ADVANCE (optimized parameter)
Singling out				
Distance to the closest	Median distance between each synthetic data point and its closest reference data point	>0.2	0.31	0.30
Distance to the closest ratio	Median of the ratio of distances between each synthetic data point and its closest and second-closest reference data points	>0.3	0.81	0.60
Linkability, %				
Column direct match protection	Minimum probability that a variable could be used as a direct identifier	>50	84.8	90.9
Row direct match protection	Percentage of synthetic data points that are identical to reference data points	>90	100	100
Inference				
Median local cloaking	Median number of avatars more similar to the reference data point of a patient than its own avatar	>5	3	6
Hidden rate, %	Probability of erroneous distance-based matching	>90	85.0	93.2
Categorical hidden rate, %	Probability of erroneous distance-based matching based on categorical variables only	>90	98.4	98.0

### Dataset Selection

From both RCTs, we selected the synthetic dataset that replicated the primary and secondary efficacy end points best while having a satisfactory level of privacy. The utilities of the datasets replicating all reported statistical test conclusions were inspected individually. In cases of equivalent utilities, the dataset with the highest privacy was preferred. In cases where no dataset replicated all the end points, the replications of the noncommercial arm end points were neglected. If still insufficient, the replication of the primary absolute and relative end points (ie, the relapse activity) was prioritized, followed by T2 MRI activity, CDW, and finally GdE MRI activity, with priority given to relative over absolute secondary end points.

### Data and Code Availability

The reference datasets may be shared upon request from Merck and Biogen. The placebo arms of the 2 selected synthetic datasets have been made publicly available as open access on the Figshare platform [50] with the approval of Merck and Biogen, although these approvals were not strictly necessary from a regulatory point of view. The code is available as R and Python notebooks at GitLab [51]. Multitable simulated versions have been rebuilt according to the original CDISC formats for educational purposes.

### Ethical Considerations

The research was conducted under the consortium agreement of the ANR-21-RHUS-0014 PRIMUS project and the MR004 data processing regulation framework of the French Personal Data Regulatory Commission (Commission nationale de l’informatique et des libertés; CNIL). It was approved by the institutional review board of Nantes University (reference 09072024). According to French law, this study was covered by the written consent for the primary studies and the information for further research use. The deidentified datasets were transferred after privacy-enhancement processes by Merck and Biogen.

## Results

### Robust Utility for the Primary End Points

**Figure 2.**
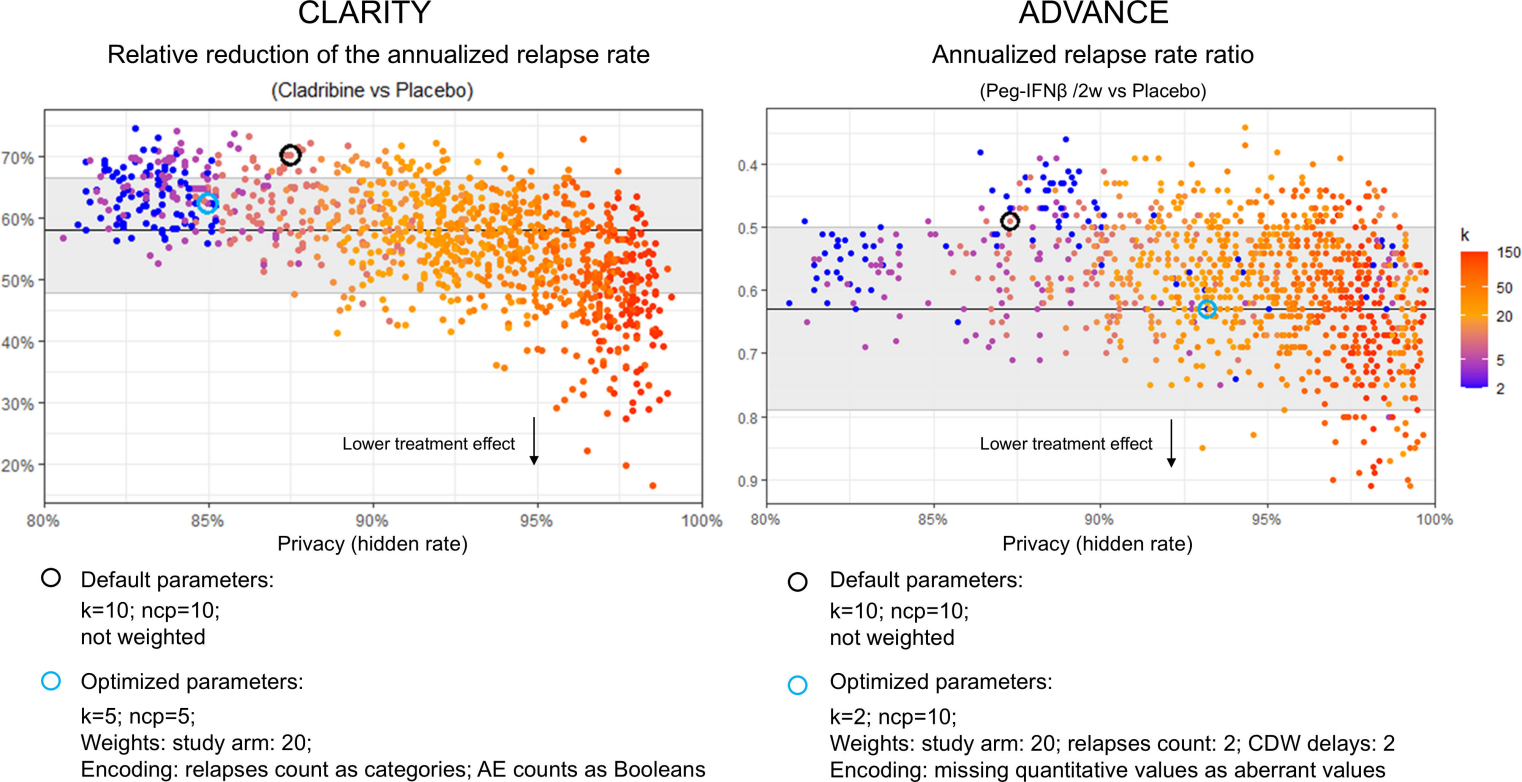
Robustness of the primary end point replications and privacy. Each point represents a generated synthetic dataset (1080 datasets per RCT). Privacy is expressed by the hidden rate, which reflects the probability of failure of a distance-based membership inference attack. The reported estimations of the primary end points are plotted with their 95% CIs (horizontal lines and gray areas). Among the 1080 generated datasets, 813 (75.3%) were within the reported 95% CI for CLARITY and 871 (80.6%) for ADVANCE. Higher privacy tended to lower the inferred treatment effect, likely reflecting the loss of statistical signal between the trial arms. The 2 selected datasets with optimized parameters are highlighted, as are the 2 generated with default configurations. AE: adverse event; CDW: confirmed disability worsening; ncp*:* number of principal components; Peg-IFNβ /2w: peginterferon beta 1 dose every 2 weeks; RCT: randomized clinical trial.

We generated 2160 synthetic datasets with varying parameter configurations, half using CLARITY and half using ADVANCE as reference datasets (Figure 1). Despite the complexity of the ADVANCE study design, only a few individual observations had to be postprocessed in some datasets for the study design to remain consistent. The missing data patterns due to attrition were well replicated, although the number of patients per arm was not necessarily as balanced as after true randomization (Figures S4 and S5 in Multimedia Appendix 1). The primary end point estimates were robustly replicated across the different configurations (Figure 2). The estimate of CLARITY was within the reported 95% CI in 783 of the 1080 datasets (72.5%), always with significant *P* values. The estimate of ADVANCE was within the reported 95% CI in 876 of the 1080 datasets (81.1%), with 873 (80.8%) of them having a significant *P* value.

### Robust Privacy

**Figure 3.**
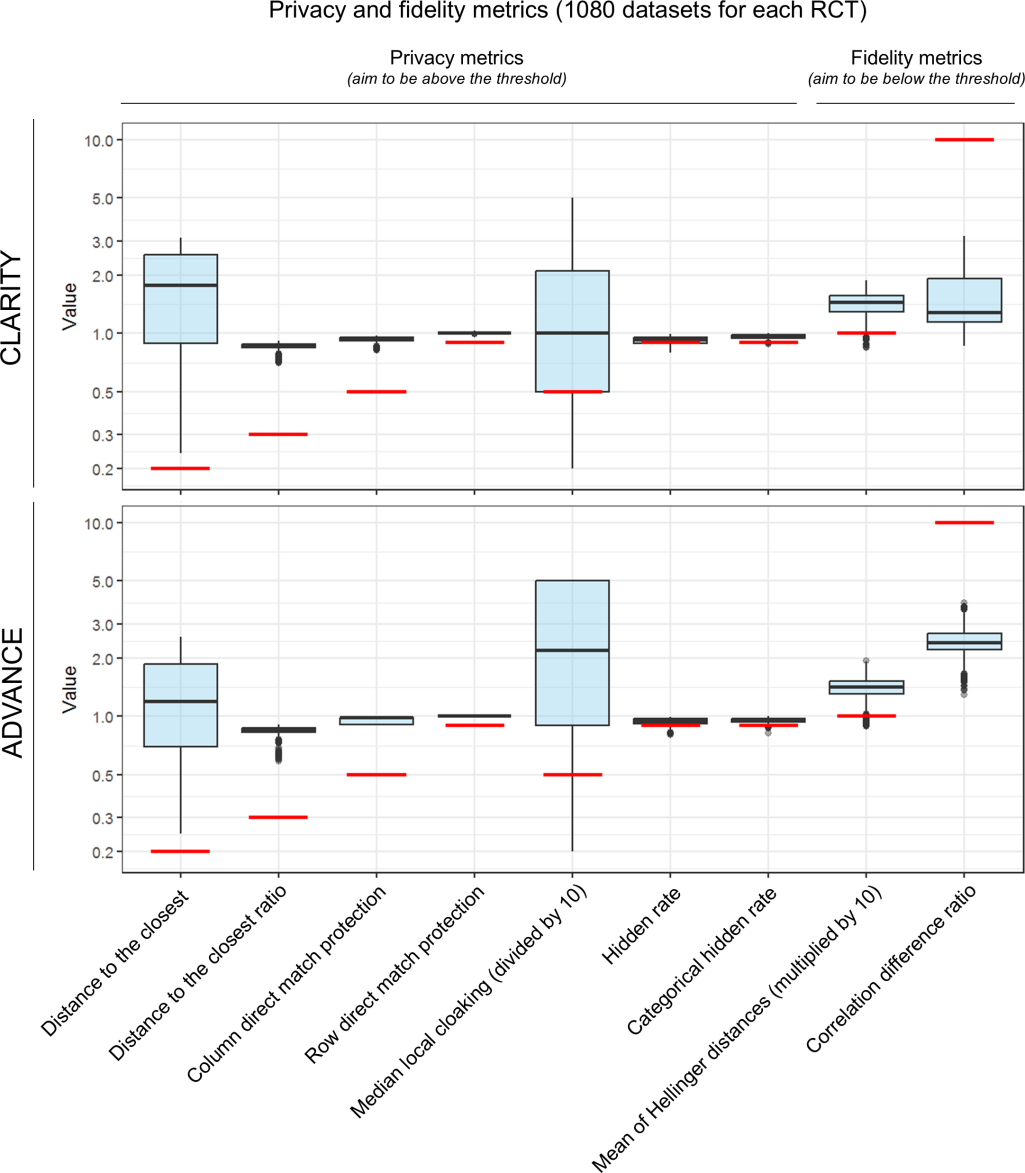
Privacy and fidelity metrics distributions of all generated datasets (1080 per randomized clinical trial). The boxes show the quartiles and the median of the values (whiskers represent quartiles ±1.5×IQR). The recommended thresholds by the software editor are plotted as red lines. Privacy metrics were expected to be above the threshold (first 7 metrics), and fidelity metrics were expected to be below the threshold (last 2 metrics). For readability, we scaled the values of some metrics and plotted those expressed in percentages as proportions. The median local cloaking is capped by the software above 50. The hidden rate, the categorical hidden rate, and mean of Hellinger distances were the metrics most difficult to pass relative to the recommended thresholds. RCT: randomized clinical trial.

Most of the 2160 generated datasets had privacy metrics passing the software editor’s recommendations (Figure 3; Tables1  2 for the numerical values). Only 4 had one avatar that was, by chance, identical to a reference data point (ie, row direct match). The distance of the avatars to the closest reference data point assesses the dispersion of the synthetic data points relative to the set of reference data points: the higher, the better the privacy. It was above 0.2 for all of the generated datasets, which is the recommended threshold by the avatars software editor (Table 1). The HR, the categorical HR, and the mean of Hellinger distances were the metrics most difficult to pass the recommended thresholds. We focused the rest of the report on HR. All 2160 generations had an HR above 80% (Figure 2). The HR increased in the postprocessed datasets whose privacies were assessed with the default encoding of all variables and unweighted FAMD projections (not shown). Overall, this shows the robustness of the avatars technique regarding privacy.

**Table 2.** Fidelity metrics of the selected datasets generated with optimized parameters.

Fidelity metric	Definition	Recommended target by Octopize (indicative)	CLARITY (optimized parameters)	ADVANCE (optimized parameters)
Mean of Hellinger distances	Mean of the Hellinger distances of each variable	<0.10	0.10	0.09
Correlation difference ratio, %	Average of the absolute variations of Pearson correlations	<10	2.52	1.49

### Synthetic Dataset Selection in the Context of a Privacy-Fidelity Trade-Off

**Figure 4.**
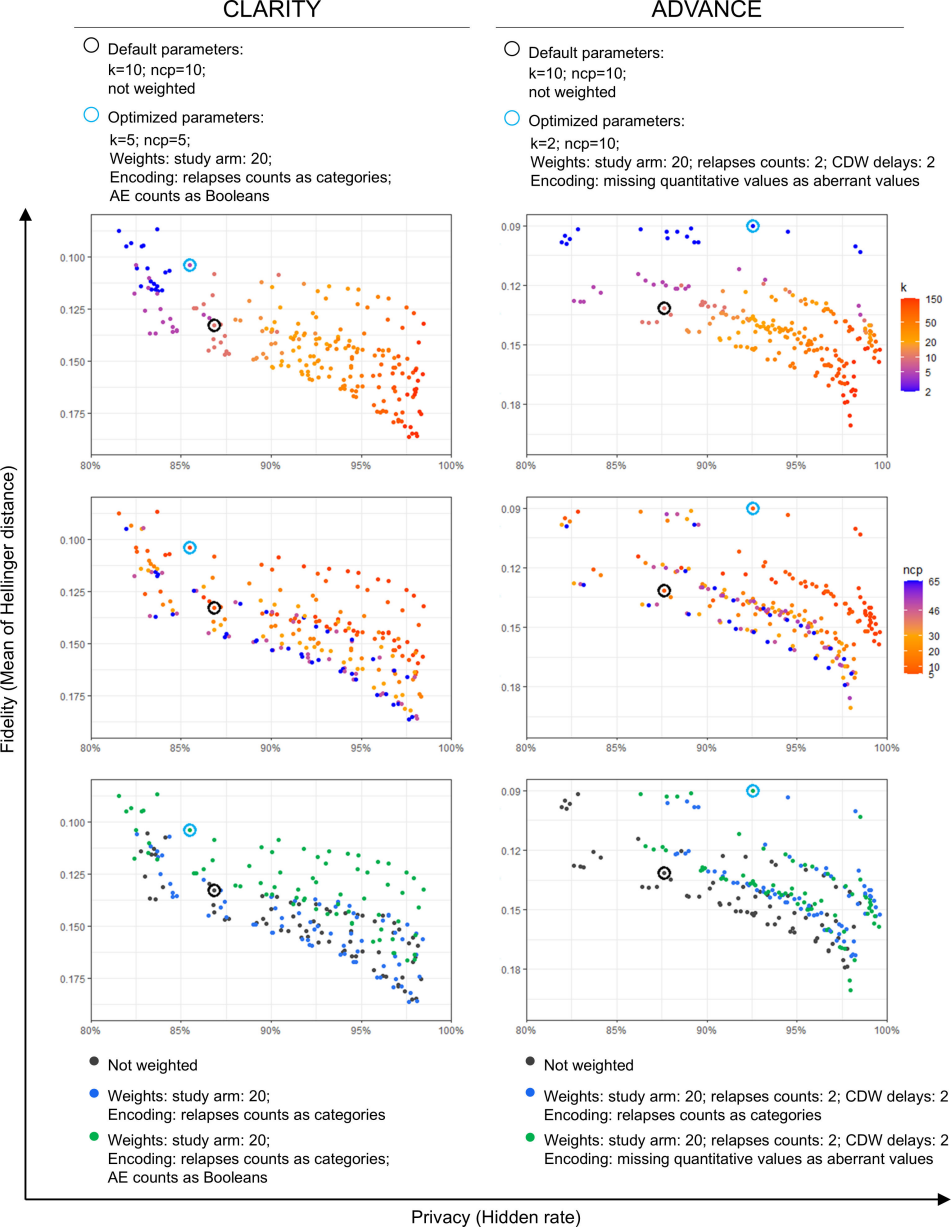
Privacy-fidelity trade-off. Each point represents the average metrics of the 5 generations with a given parameter configuration but different random states (216 groups per RCT). Privacy is expressed by the hidden rate, assessing the probability of failure of a distance-based membership inference attack. Fidelity is expressed as the mean of the Hellinger distances between the univariate distributions. Weighting and encoding some variables differently could optimize the trade-off, as reflected by the generation of datasets closer to the “sweet spot” in the upper right corner (high privacy and high fidelity). The 2 selected datasets are highlighted, as are the 2 generated with default configurations. Greater fidelity did not automatically improve utility, as reflected by the position of the selected datasets. AE: adverse event; CDW: confirmed disability worsening; ncp: number of principal components; RCT: randomized clinical trial.

The assessment of privacy, fidelity, and utility showed a privacy-fidelity trade-off (Figure 4). We assessed fidelity with the mean of the Hellinger distances between the univariate distributions. Small k values increased fidelity while decreasing privacy. A small ncp value increased fidelity with few effects on privacy. Weighting and encoding some variables differently could optimize the trade-off, as reflected by the generation of datasets closer to the “sweet spot” with both high fidelity and privacy. A better fidelity did not automatically improve utility. For CLARITY, four datasets (0.4%) replicated all primary and secondary efficacy end points. For ADVANCE, no dataset replicated all primary and secondary efficacy end points for the 2 tested regimens, but 14 did when neglecting the noncommercial regimen (1.3%). For CLARITY, we selected the dataset with the best replication of absolute estimates, generated with k=5, ncp=5, weighting of the study arm by 20, and encoding of relapse counts as categories (0, 1, 2, and 3 or more) and AE counts as Booleans (none vs any). Such encoding was reverted at postprocessing before replicating the RCT analysis, but yielded some granularity loss. For ADVANCE, we selected the dataset generated with k=2, ncp=10, weighting of the study arm by 20, relapse counts and CDW delays by 2, and missing quantitative values encoded as aberrant negative values. The selected dataset from CLARITY had a median LC of 3 and an HR of 85.0%; the one from ADVANCE had a median LC of 6 and an HR of 93.2% (Table 1). We focus the rest of the report on both selected datasets (referred to as “optimized”) and 2 datasets generated with default parameters (k=10; ncp=10; not weighted) and the third random state.

### Good Fidelity at the Population Level Despite Alterations in Variable Distributions

**Figure 5.**
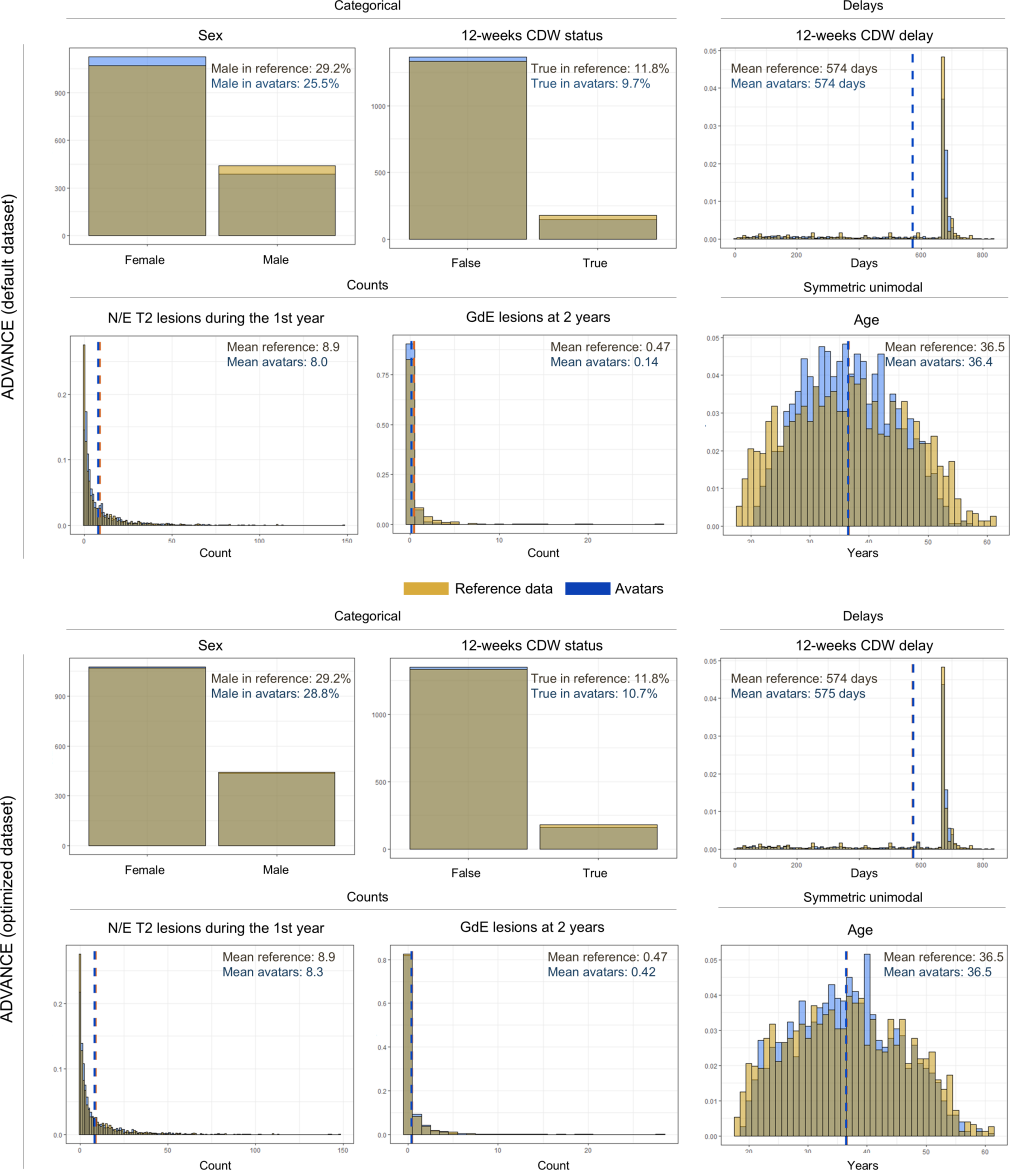
Impairment of univariate distributions. Comparisons of illustrative univariate distributions for the default and selected datasets generated from ADVANCE (top and bottom panels, respectively). Means are plotted as dashed lines. The avatars technique altered the distributions to varying degrees depending on the type of variable. The largest effects were observed for count distributions. CDW: confirmed disability worsening; FAMD: factorial analysis of mixed data; GdE: gadolinium-enhancing; N/E: new or enlarging.

**Figure 6.**
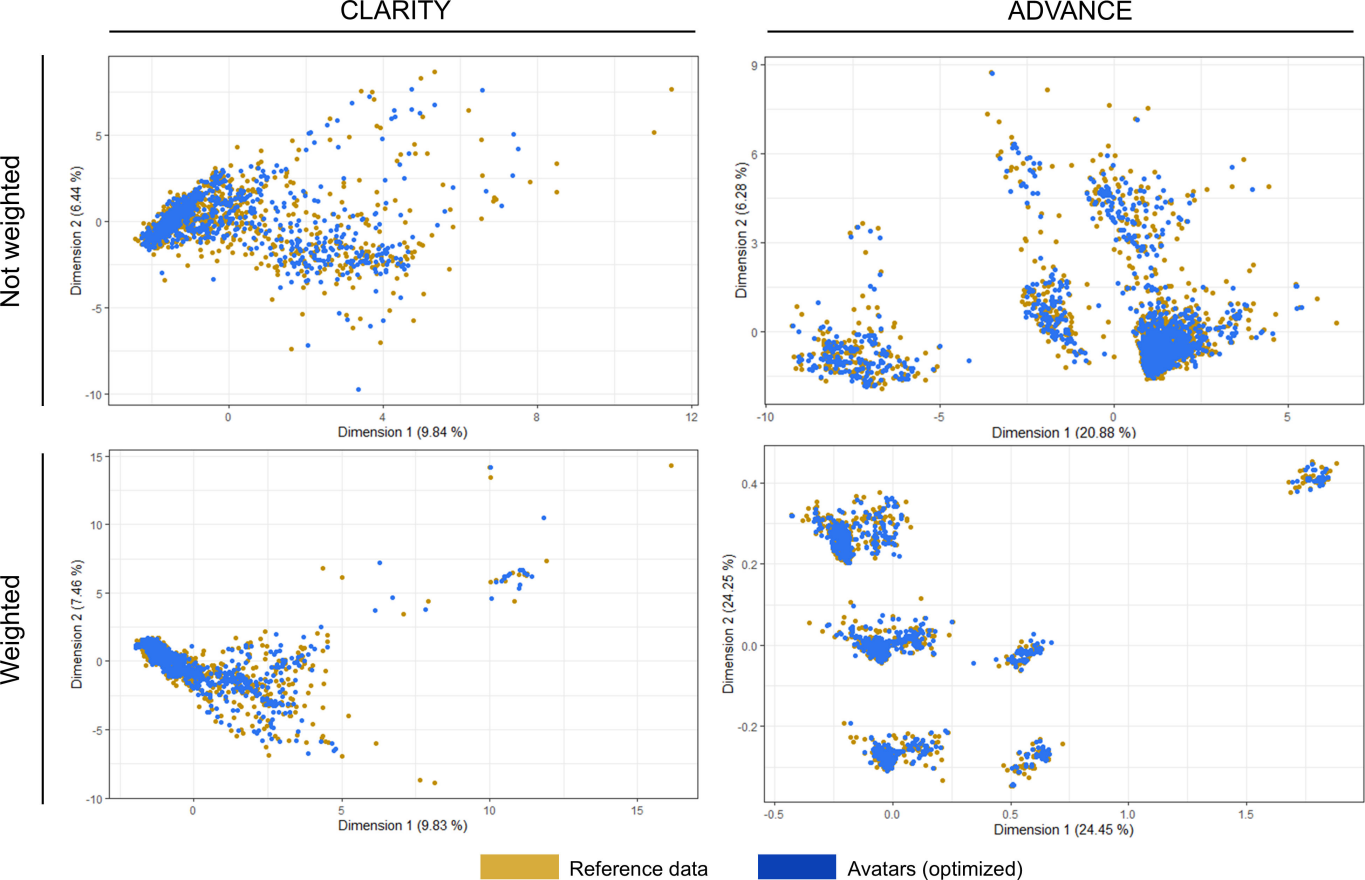
Comparison of the factor analysis of mixed data projections of the selected datasets with or without applying the weights of the respective parameter configuration. As a result of the primary design of the avatars as an anonymization technique, the avatar data points clustered in higher-density regions, which is less reidentifiable. FAMD: factor analysis of mixed data.

The mean of Hellinger distances was 0.10 and 0.09 for the selected datasets from CLARITY and ADVANCE, respectively (Table 2). The effects of the avatar method on variable distributions were consistent across all generated datasets, modulated only by different parameter configurations (Figure 5). The distributions of categorical variables were the most preserved, with a tendency to amplify class imbalances. The distributions of quantitative variables tended to be narrowed and normalized, but their means were similar if they had a limited skewness. Of note, many distributions, especially MRI lesion counts, were skewed, with 0 being the majority value and many outliers on the right tail. As a result of the privacy-by-design approach, the avatars of the outliers were drastically recentered toward high-density regions in the synthetic dataset, as shown by the weighted FAMD projections (Figure 6), which tended to decrease the average absolute counts. The most affected variable was the count of GdE lesions at 2 years in ADVANCE. Its average was reduced by about a factor of 3 in the default dataset (0.47 to 0.14), which could be mitigated with the optimized configuration. Bivariate distributions were similar (Figure S2 in Multimedia Appendix 1), as were the missing data patterns (Figure S3 in Multimedia Appendix 1).

### The Utility for Multiple End Points Needs Optimization

**Figure 7.**
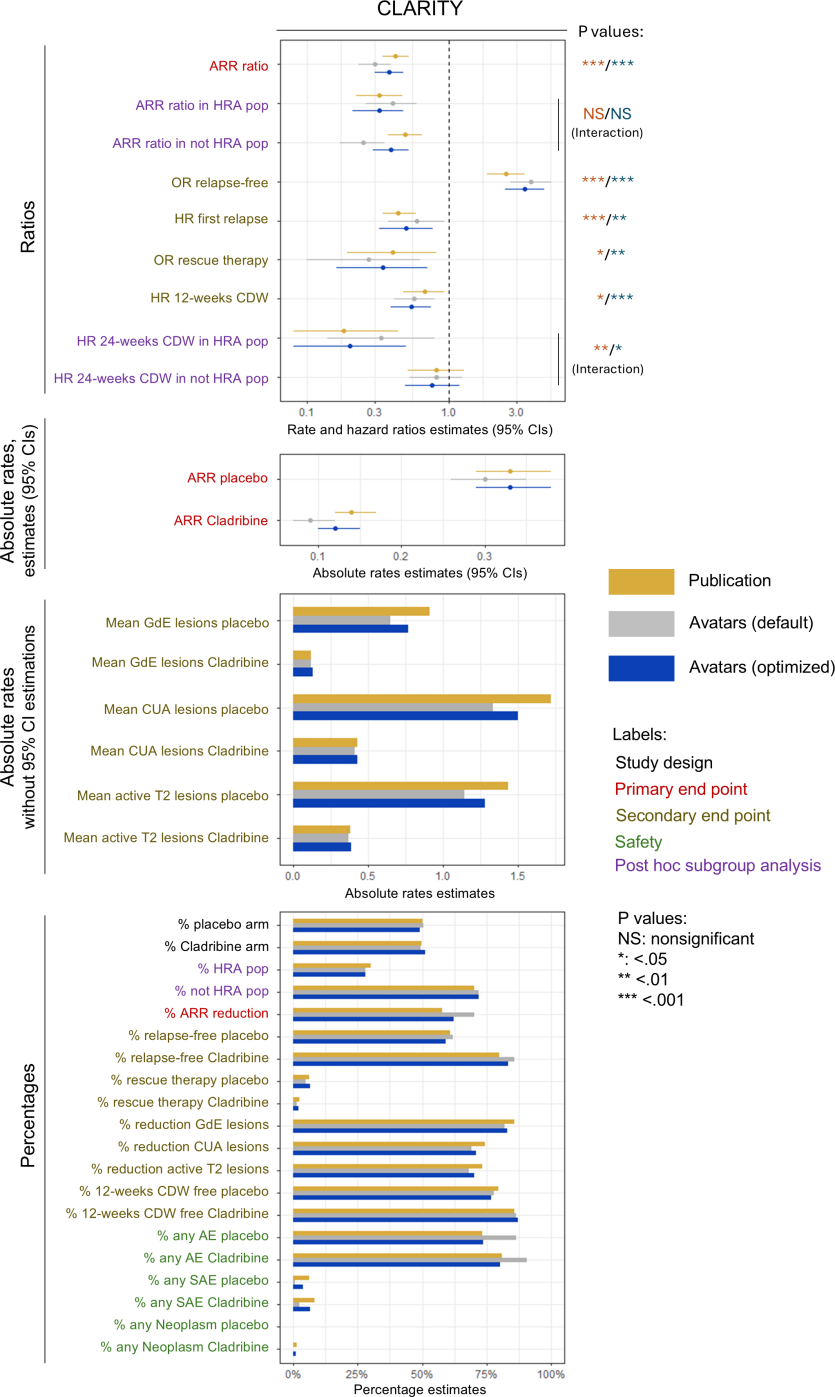
Utility assessment of the default and selected datasets from CLARITY. All end points were analyzed over 2 years and against placebo. The analyses were adjusted for covariates as reported. The *P* values of the subgroup analysis end points correspond to the interaction tests. Only the most important types of adverse events are displayed. The optimized configuration mitigated the limitations of the avatars technique observed with the default configuration, especially for the replication of absolute rates and safety outcomes. AE: adverse event; ARR: annualized relapse rate; CDW: confirmed disability worsening; CUA: combined unique active; GdE: gadolinium-enhancing; HR: hazard ratio; HRA: high relapse activity (ie, 2 relapses or more during the year preceding the study baseline); OR: odds ratio; SAE: severe adverse event.

While most generations replicated the primary end point of the respective RCT, replicating all secondary end points was more challenging (Figures7  8). Generations with default parameters replicated most relative end points but tended to shift absolute end points due to the amplification of class imbalance by the avatars technique, increasing the percentages of the most represented classes and decreasing those of the minority classes. ARR and lesion rates were highly sensitive to the average shift of count variables. This limitation could be mitigated by optimizing the parameters, especially the weighting and encoding of some variables. The replications of the flowcharts and tables of both RCT reports are presented in Figures S4 and S5, Tables S3-S5 and S6‐S7 in Multimedia Appendix 1.

**Figure 8.**
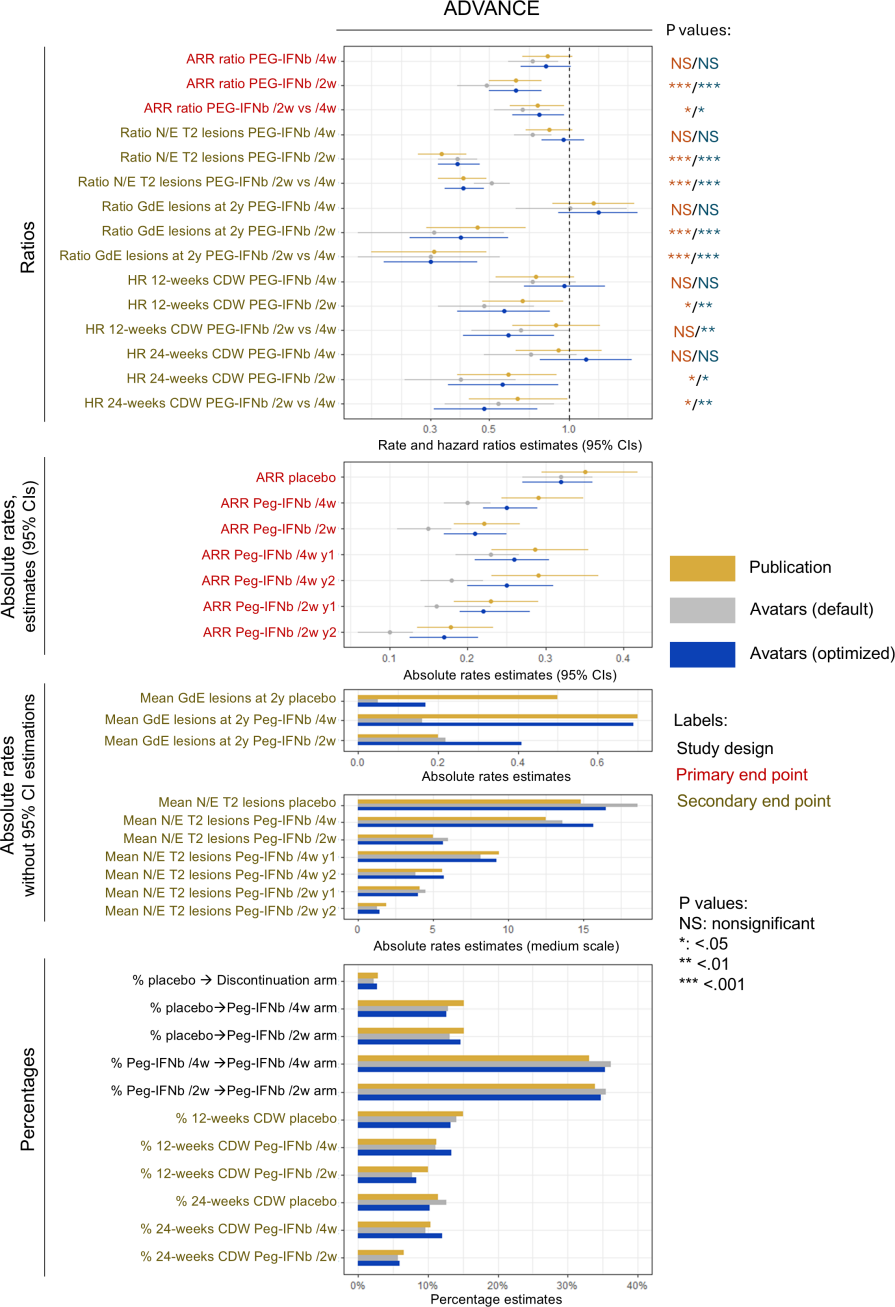
Utility assessment of the default and selected datasets from ADVANCE. All end points were analyzed over 2 years and against placebo unless specified (eg, year 1 and year 2). The analyses were adjusted for covariates as reported. All primary end points and all relative secondary end points were successfully replicated for the commercial “1 dose per 2 weeks” regimen. The optimized configuration mitigated the limitations of the avatars technique observed with the default configuration, especially for replication of absolute rates. ARR: annualized relapse rate; CDW: confirmed disability worsening; GdE: gadolinium-enhancing; HR: hazard ratio; N/E T2 lesions: new or enlarging T2 lesions; Peg-IFNb/2w: peginterferon beta-1 dose every 2 weeks; Peg-IFNb/4w: peginterferon beta-1 dose every 4 weeks.

For CLARITY (Figure 7), we pushed the assessment of specific utility up to the replication of interaction tests in a post hoc subgroup analysis in patients with high relapse activity (ie, 2 or more relapses the year before the study baseline) [45]. The alteration in univariate distributions by the avatars method suggested that subgroup analyses would be harder to replicate, but the selected dataset managed to do so. These post hoc subgroup analyses were critical for the market approval of cladribine in this subpopulation, as the initial submission for the whole relapsing-remitting MS population had been withdrawn due to safety concerns about the risk of neoplasm (6 vs 0 patients in the real dataset). The safety end points were very sensitive to the skewness of count distributions, such that the proportions of patients with serious AEs were drastically reduced in the default dataset. Encoding AEs as Booleans mitigated this and also replicated the contrast of neoplasm incidence (5 avatars with cladribine vs 0 with placebo). The replication of the RCT report tables is provided in Tables S3-S5 in Multimedia Appendix 1.

For ADVANCE (Figure 8), the complex design aimed to compare MS activity during the second year against the first year of treatment to assess the run-in (ie, delay of action) of Peg-IFNβ. Indeed, the selected dataset and the one generated with default parameters replicated the decrease of the ARR during year 2 with the “1 dose per 2 weeks” regimen, while only the optimized dataset replicated the stability of the ARR with the “1 dose per 4 weeks” regimen. In the selected dataset, the only end point that could not be replicated was the 12-week CDW hazard ratio estimate between both tested regimens and the 24-week CDW hazard ratio estimate for the noncommercial regimen. The first was outside the reported 95% CI with a *P* value that became significant, while the second was in the wrong direction. The replicability of the absolute GdE lesion count was poor, whatever the configuration. This limitation was likely associated with the skewness of this variable distribution, which was essentially composed of outliers (Figure 5). The replication of the RCT report tables is provided in Tables S6 and S7 in Multimedia Appendix 1.

### The Utility for Other Downstream Tasks is Not Guaranteed

Performing prediction tasks on synthetic data yielded better performances than when performed on reference data (Tables3  4). The higher the performance of the reference model, the more important the increase in performance of the test model. This indicated a simplification of the data patterns in the synthetic datasets, which is consistent with the normalization of univariate distributions and the decrease of outliers (Figure 5). This interpretation was reinforced by the similar or better performances of the reference models when evaluated on the synthetic datasets. Likewise, we controlled for overfitting on unrealistic patterns in the synthetic datasets by evaluating the test models on the reference datasets. The test models had similar or better performances on the reference datasets than the reference models, which suggested a regularizing effect of the synthetic data. Yet, the difference in prediction performances could also result from the amplification of class imbalance. Overall, these necessitate that the utility assessment of synthetic datasets prioritize the end points, as their replicabilities are uneven and may be conditioned by the characteristics of the reference dataset. The synthetic data generation may be optimized toward a given purpose by weighting some variables or encoding them differently.

**Table 3.** Predictive capacity of the datasets generated from CLARITY.

Experiment and metric	Reference data, estimate (95% CI)	Synthetic data (default), estimate (95% CI)	Synthetic data (optimized), estimate (95% CI)
Reference model on reference data and test model on synthetic data			
Relapse activity over 2 years			
AUC^a^	0.57 (0.48-0.66)	0.76 (0.70-0.82)^b^	0.75 (0.67-0.82)^b^
Accuracy	0.74 (0.69-0.80)	0.77 (0.72-0.82)	0.75 (0.69-0.80)
MRI^c^ activity over 2 years			
AUC	0.76 (0.69-0.82)	0.80 (0.73-0.87)	0.89 (0.84-0.93)^b^
Accuracy	0.75 (0.69-0.80)	0.82 (0.77-0.86)^b^	0.87 (0.83-0.91)^b^
Reference model on synthetic data			
Relapse activity over 2 years			
AUC	—^d^	0.76 (0.69-0.83)^b^	0.71 (0.63-0.78)^b^
Accuracy	—	0.76 (0.71-0.82)	0.73 (0.67-0.78)
MRI activity over 2 years			
AUC	—	0.88 (0.82-0.93)^b^	0.88 (0.82-0.93)^b^
Accuracy	—	0.85 (0.81-0.89)^b^	0.83 (0.78-0.87)^b^
Test model on reference data			
Relapse activity over 2 years			
AUC	—	0.73 (0.66-0.81)^b^	0.70 (0.61-0.78)^b^
Accuracy	—	0.73 (0.67-0.79)	0.76 (0.71-0.82)
MRI activity over 2 years			
AUC	—	0.78 (0.71-0.84)	0.84 (0.78-0.89)^b^
Accuracy	—	0.77 (0.72-0.82)	0.81 (0.75-0.85)^b^

a AUC: area under the receiver operating characteristic curve.

b Values outside the CI95% of the reference model performances

c MRI: magnetic resonance imaging.

d Not applicable.

**Table 4.** Predictive capacity of the datasets generated from ADVANCE.

Experiment and metric	Reference data, estimate (95% CI)	Synthetic data (default), estimate (95% CI)	Synthetic data (optimized), estimate (95% CI)
Reference model on reference data and test model on synthetic data			
Relapse activity over year 1			
AUC^a^	0.60 (0.53-0.67)	0.78 (0.73-0.84)^b^	0.77 (0.70-0.82)^b^
Accuracy	0.79 (0.75-0.83)	0.84 (0.81-0.88)^b^	0.82 (0.79-0.86)
MRI^c^ activity over year 1			
AUC	0.78 (0.73-0.83)	0.87 (0.81-0.92)^b^	0.89 (0.85-0.92)^b^
Accuracy	0.79 (0.75-0.83)	0.90 (0.86-0.93)^b^	0.86 (0.82-0.89)^b^
Reference model on synthetic data			
Relapse activity over year 1			
AUC	—^d^	0.79 (0.74-0.84)^b^	0.87 (0.83-0.91)^b^
Accuracy	—	0.80 (0.77-0.84)	0.83 (0.80-0.87)
MRI activity over year 1			
AUC	—	0.91 (0.88-0.95)^b^	0.95 (0.93-0.97)^b^
Accuracy	—	0.90 (0.86-0.93)^b^	0.88 (0.85-0.91)^b^
Test model on reference data			
Relapse activity over year 1			
AUC	—	0.72 (0.65-0.78)^b^	0.79 (0.73-0.84)^b^
Accuracy	—	0.77 (0.73-0.81)	0.82 (0.79-0.86)
MRI activity over year 1			
AUC	—	0.80 (0.75-0.85)	0.88 (0.84-0.92)^b^
Accuracy	—	0.79 (0.75-0.83)	0.85 (0.81-0.88)^b^

a AUC: area under the ROC curve.

b Values outside the CI95% of the reference model performances

c MRI: magnetic resonance imaging.

d Not applicable.

## Discussion

### Principal Findings

While a report of the avatars technique already provided proof of concept that a synthetic dataset could reproduce the primary end point [37], our study showed that it is possible to generate synthetic datasets replicating most absolute and relative end points reported in the publications while implementing the regulatory guidance about anonymization. The method proved robust for privacy and the replication of the primary end point, but finding a satisfactory utility required optimization. This optimization process is analogous to the development and selection of machine learning models after searching for the optimal algorithm family and hyperparameters. In our use case, the explicit privacy assessment allowed us to legally qualify the synthetic datasets as nonpersonal data and share them as open datasets. Satisfactory utility was even achieved with the complex study design of ADVANCE, which suggests the ability of the avatars technique to capture the information of a wide range of RCTs and complex datasets in other fields.

### Limitations

This study did not compare the avatars to some benchmark algorithms. The first report of the avatars method performed such an analysis against Synthpop and CT-GAN and showed that the avatars outperformed them in replicating the primary end point of an RCT and a cohort of real-world data [37]. Although not performed on the same reference datasets, we considered this result sufficiently established to focus the efforts and the analysis of this study on the privacy and replication of the multiple end points an RCT may have.

It remains that the whole granularity of the reference datasets could not be captured, which would be a requirement to use the synthetic dataset as an external comparator. Such external comparison has been performed in the CHAMPION trial in neuromyelitis optica spectrum disorder, a rare and aggressive disease, to evaluate ravulizumab while avoiding exposing patients to a placebo [7]. For educational purposes, we also provided a simulation of a rebuilt version of the synthetic datasets into the CDISC standards, as received by Merck and Biogen. Our results showed that encoding the reference data in a more aggregated fashion (relapse counts as 4-level categorical variables, AEs count as Booleans) improved the utility regarding the corresponding end points. This aggregation could have been pushed further at the cost of a narrowed intended use of the generated datasets. This and the partial data transfer by the industrials limited the granularity that could be captured by the avatars technique.

Since the avatars technique has been primarily developed as an anonymization technique, it tends to recenter the data points in the latent space and alter the univariate distributions because minoritarian profiles and outliers are easier to reidentify. This is likely to limit the use of the synthetic datasets for exploratory subgroup analysis in populations defined by several criteria or as external synthetic control arms, should significant subgroup matching with the real experimental arm be necessary. Furthermore, the fact that better fidelity did not automatically result in better utility highlights that the assessment of a synthetic dataset cannot be agnostic of the intended use. As such, post hoc analysis of synthetic datasets can only be hypothesis-generating.

As suggested by the alternative variable weighting and encodings, our results could be improved by complexifying the data preprocessing (eg, normalizing count data with log transforms) and the synthetic dataset generation (eg, one generation per study arm). In truth, the parameter space with alternative weighting, encoding of variables, and random states could not be explored exhaustively because of computational cost considerations.

The selected synthetic datasets had median LCs and HRs below the targets generally recommended by the software editor (Table 1). These targets are only indicative. No technical consensus exists about the required privacy metrics and their acceptable levels. In our specific use case, one has to take into account the combination of other privacy-enhancing processes such as deidentification, time shifting, exclusion of any medico-administrative variable to retain only specialized variables about MS (ie, data minimization), the aggregation of data into an integrated analysis-ready table, and the increase of HR after postprocessing. This suggests that the privacy-fidelity and privacy-utility trade-offs of synthetic data generation should be evaluated on a case-by-case basis.

### Perspectives

The privacy-fidelity trade-off highlighted by the 2160 datasets we generated (Figure 4) and the uneven utilities (Figures7  8) are both a limit of the agnostic exploration of synthetic datasets and a perspective for usage control over the data value chain. Beyond the risk of patient reidentification from individual observations, the owner of a reference database may be concerned by the loss of control over the information of a dataset, should a synthetic dataset have a high and broad utility. According to the intended usage of the synthetic data, the generation may be parametrized or the dataset selected to favor utility or privacy and specific variables. As such, a dedicated study would be required to analyze the performance gain of the predictive models trained on synthetic data (Tables3  4).

In contrast to the data-centric approach of synthetic data, the dominant trend in sharing information for medical research is to share calibrations of parametric models. Federated learning is the archetypal framework for developing deep learning models with sensitive data [52]. Both approaches have been compared operationally, with significantly faster processes when sharing synthetic data [53]. Still, even if the sensitive data is not shared, the privacy of the model learned from them remains questionable [18-20]. Therefore, both approaches could supplement one another, with federated learning enabling data owners to enforce their control rules, while synthetic data would address the privacy risk and augment datasets for a given use or context.

### Conclusion

We generated synthetic RCT datasets and selected 2 for release as open datasets with a satisfactory trade-off between privacy and utility. To the best of our knowledge, it is the first report of virtual trials replicating all reported efficacy end points for the placebo and approved regimen arms of several RCTs. The synthetic datasets may be used for various exploratory uses, but the information captured is insufficient for a complete indirect treatment comparison. The privacy-fidelity trade-off and the uneven utility show that synthetic data generation has to be purpose-driven, rather than agnostic of the intended use. Besides the privacy enhancement of synthetic datasets, their limited validity for unintended uses provides usage control to the owner of the reference data.

## Supplementary material

10.2196/71297 Multimedia Appendix 1 Additional tables and figures.

## Abbreviations

AE: adverse event
ARR: annualized relapse rate
CDISC: Clinical Data Interchange Standards Consortium
CDW: confirmed disability worsening
CT-GAN: Conditional Transformation-Generative Adversarial Network
FAMD: factorial analysis of mixed data
GAN: generative adversarial network
GdE: gadolinium-enhancing
HR: hidden rate
IPD: individual patient data
LC: local cloaking
MRI: magnetic resonance imaging
MS: multiple sclerosis
Peg-IFNβ: peginterferon beta
RCT: randomized clinical trial
