# Supplementary material for: Privacy-by-Design Approach to Generate Two Virtual Clinical Trials for Multiple Sclerosis and Release Them as Open Datasets: Evaluation Study
Source: J Med Internet Res. 2025 Oct 1;27:e71297. doi: 10.2196/71297 (PMC12488035; doi:10.2196/71297)
Supplement: Multimedia Appendix 1 [file jmir-v27-e71297-s001.docx]

# Table S1. Dictionary of the minimal dataset of CLARITY. The main study lasted 2 years and was followed by an extension study. CDW: confirmed disability worsening; CUA: Combined Unique Active; EDSS; Expanded disability status scale; GdE: Gadolinium-enhancing.

| \| Variable \| Default encoding \| Definition \| \| --- \| --- \| --- \| \| index_itt_treatment \| factor \| Intention-to-treat treatment \| \| horizon_mainStudyDisposition_2y \| factor \| Main study disposition \| \| index_age \| num \| Age at baseline \| \| index_sex \| factor \| Sex \| \| index_weight \| num \| Weight at baseline \| \| index_diseaseDuration \| num \| Years since the disease onset \| \| index_edss \| num \| Baseline EDSS \| \| index_nbT1GdBrain \| num \| Number of GdE lesions at baseline \| \| index_volT2HyperBrain \| num \|  \| \| horizon_nbRelapses_toMainEnd \| num \| Number of relapses during the main study \| \| horizon_exposure_0_2y \| num \| Follow-up duration \| \| index_geographicRegion \| factor \| Geographic region \| \| past_diff_nbRelapses_1y \| num \| Number of relapses the previous years \| \| horizon_rescueTherapy_0_2y \| factor \| Rescue therapy received \| \| horizon_delay_relapse \| num \| Survival delay to the first relapse \| \| horizon_status_CDW3m \| factor \| Survival status for 12-week-CDW \| \| horizon_delay_CDW3m \| num \| Survival delay for 12-week-CDW \| \| horizon_status_CDW6m \| factor \| Survival status for 24-week-CDW \| \| horizon_delay_CDW6m \| num \| Survival delay for 24-week-CDW \| \| horizon_nbT1GdBrain_mean_0_2y \| num \| Mean number of GdE lesions per MRI scan \| \| horizon_nbActiveT2HyperBrain_mean_0_2y \| num \| Mean number of T2 active lesions per MRI scan \| \| horizon_nbCUABrain_mean_0_2y \| num \| Mean number of CUA lesions per MRI scan \| \| horizon_nbAe_toMainEnd \| num \| Number of adverse events during the main study \| \| horizon_nbMildHeadache_toMainEnd \| num \| Number of mild headache during the main study \| \| horizon_nbSevereHeadache_toMainEnd \| num \| Number of severe headache during the main study \| \| horizon_nbMildLymphocytopenia_toMainEnd \| num \| Number of mild lymphopenia during the main study \| \| horizon_nbSevereLymphocytopenia_toMainEnd \| num \| Number of severe lymphopenia during the main study \| \| horizon_nbMildNasopharyngitis_toMainEnd \| num \| Number of mild nasopharyngitis during the main study \| \| horizon_nbMildUrti_toMainEnd \| num \| Number of mild upper respiratory tract infections during the main study \| \| horizon_nbMildNausea_toMainEnd \| num \| Number of mild nausea during the main study \| \| horizon_nbSevereNausea_toMainEnd \| num \| Number of severe nausea during the main study \| \| horizon_nbSae_toMainEnd \| num \| Number of serious adverse events during the main study \| \| horizon_nbInfection_toMainEnd \| num \| Number of infections during the main study \| \| horizon_nbNeoplasm_toMainEnd \| num \| Number of neoplasms during the main study \| \| horizon_nbFatalAe_toMainEnd \| num \| Number of fatal adverse events during the main study \| |
| --- | --- | --- | --- | --- | --- | --- | --- | --- | --- | --- | --- | --- | --- | --- | --- | --- | --- | --- | --- | --- | --- | --- | --- | --- | --- | --- | --- | --- | --- | --- | --- | --- | --- | --- | --- | --- | --- | --- | --- | --- | --- | --- | --- | --- | --- | --- | --- | --- | --- | --- | --- | --- | --- | --- | --- | --- | --- | --- | --- | --- | --- | --- | --- | --- | --- | --- | --- | --- | --- | --- | --- | --- | --- | --- | --- | --- | --- | --- | --- | --- | --- | --- | --- | --- | --- | --- | --- | --- | --- | --- | --- | --- | --- | --- | --- | --- | --- | --- | --- | --- | --- | --- | --- | --- | --- | --- | --- | --- |
|  |

**Table S2.** Dictionary of the minimal dataset of ADVANCE. CDW: confirmed disability worsening; EDSS; Expanded disability status scale; GdE: Gadolinium-enhancing.

| \| Variable \| Default encoding \| Definition \| \| --- \| --- \| --- \| \| TRTREA1 \| factor \| Treatment disposition (first year) \| \| TRTREA2 \| factor \| Treatment disposition (second year) \| \| ARM \| factor \| Study arm \| \| EDSSBL \| num \| Baseline EDSS \| \| RLPS3YR \| num \| Number of relapses the 3 previous years \| \| AGE \| num \| Age at baseline \| \| YRSON_0_1y \| num \| Follow-up duration during the first study year \| \| YRSON_1_2y \| num \| Follow-up duration during the second study year \| \| nbRelapses_0_1y \| num \| Number of relapses during the first study year \| \| nbRelapses_1_2y \| num \| Number of relapses during the second study year \| \| nbNewOrGrowingT2Lesions_0_1y \| num \| Number of new or enlarging T2 lesions during the first study year \| \| nbNewOrGrowingT2Lesions_1_2y \| num \| Number of new or enlarging T2 lesions during the second study year \| \| cdw12w_status \| factor \| Survival status for 12-week-CDW \| \| cdw12w_delay \| num \| Survival delay for 12-week-CDW \| \| cdw24w_status \| factor \| Survival status for 24-week-CDW \| \| cdw24w_delay \| num \| Survival delay for 24-week-CDW \| \| T2LESBL \| num \| Number of T2 lesions at baseline \| \| nbGadoLesions_2y \| num \| Number of GdE lesions at 2 years \| \| GDLESBL \| num \| Number of GdE lesions at baseline \| \| GDLESGR \| factor \| Presence of GdE lesions \| \| SEX \| factor \| Sex \| \| RLPS1YR \| num \| Number of relapses the previous years \| \| ONSYRS \| num \| Years since the disease onset \| \| DIAGYRS \| num \| Years since the diagnosis of the disease \| \| MCDBL \| factor \| McDonald diagnostic criteria status at baseline \| |
| --- | --- | --- | --- | --- | --- | --- | --- | --- | --- | --- | --- | --- | --- | --- | --- | --- | --- | --- | --- | --- | --- | --- | --- | --- | --- | --- | --- | --- | --- | --- | --- | --- | --- | --- | --- | --- | --- | --- | --- | --- | --- | --- | --- | --- | --- | --- | --- | --- | --- | --- | --- | --- | --- | --- | --- | --- | --- | --- | --- | --- | --- | --- | --- | --- | --- | --- | --- | --- | --- | --- | --- | --- | --- | --- | --- | --- | --- | --- |
|  |

# Figure S1. Robustness for the primary endpoint replications and privacy (full version). Each point represents a generated synthetic dataset (n=1080 datasets for each RCT). Privacy is expressed by the hidden rate, assessing the probability of failure of a distance-based membership inference attack. The reported estimations of the primary endpoints are plotted with their 95% CI (horizontal lines and grey areas). Over the 1080 generated datasets, 813 (75.3%) were within the reported 95% CI for CLARITY and 871 (80.6%) for ADVANCE. Higher privacy tended to lower the inferred treatment effect, which likely reflected the pooling of statistical signals between both arms. The 2 selected datasets with optimized parameters are highlighted, as well as the 2 generated with default configurations. AE: adverse events; CDW: confirmed disability worsening; CI: confidence interval; *ncp*: number of principal components; Peg-IFNb/2w: Peginterferon beta 1 dose every 2 weeks; RCT: randomized clinical trial.

| 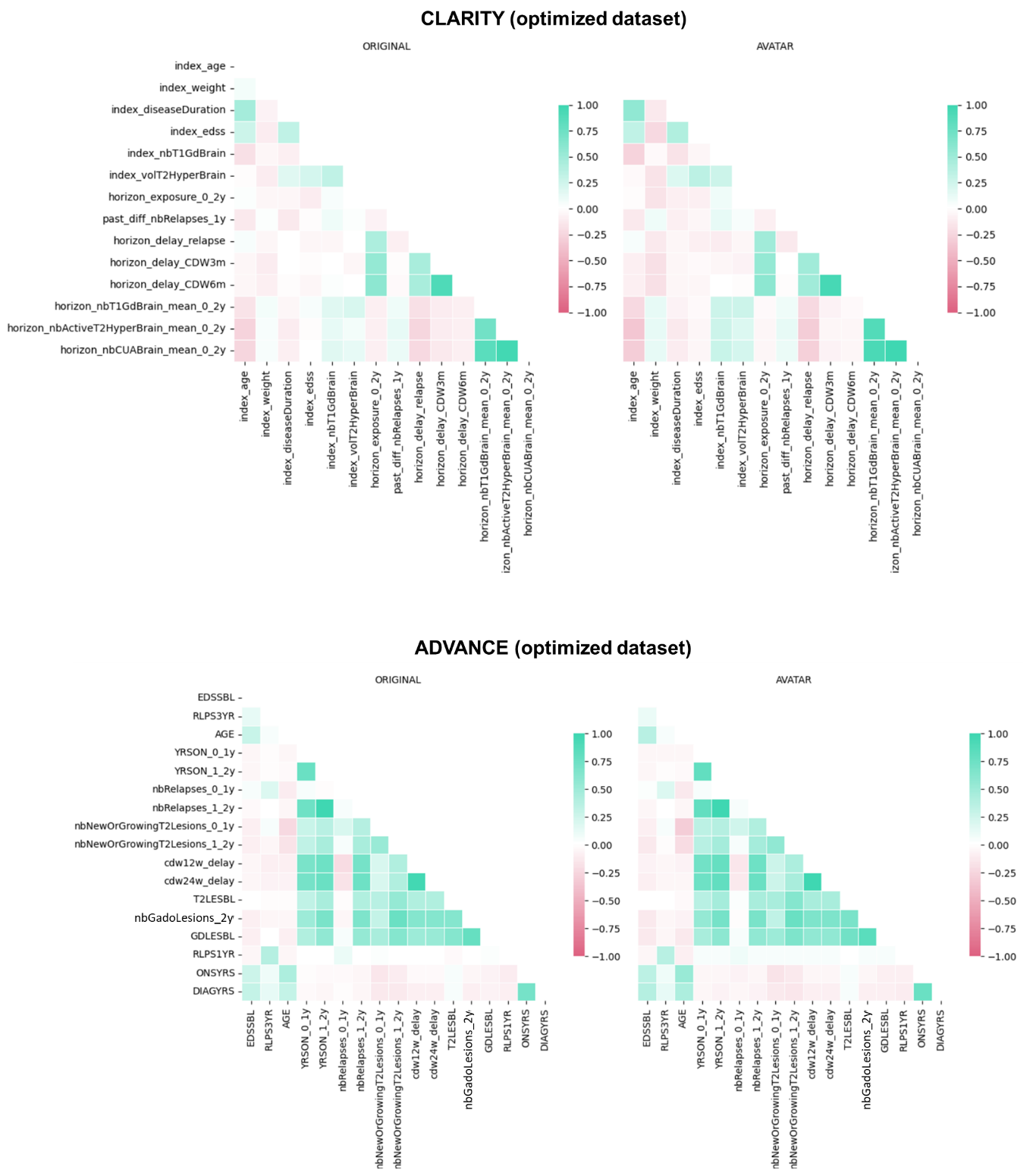 |
| --- |
| **Figure S2.** Comparison of the bivariate correlations of the quantitative variables assessed by the Pearson coefficient. The figures are extracted from the avatarization report generated by the avatars client. |

| 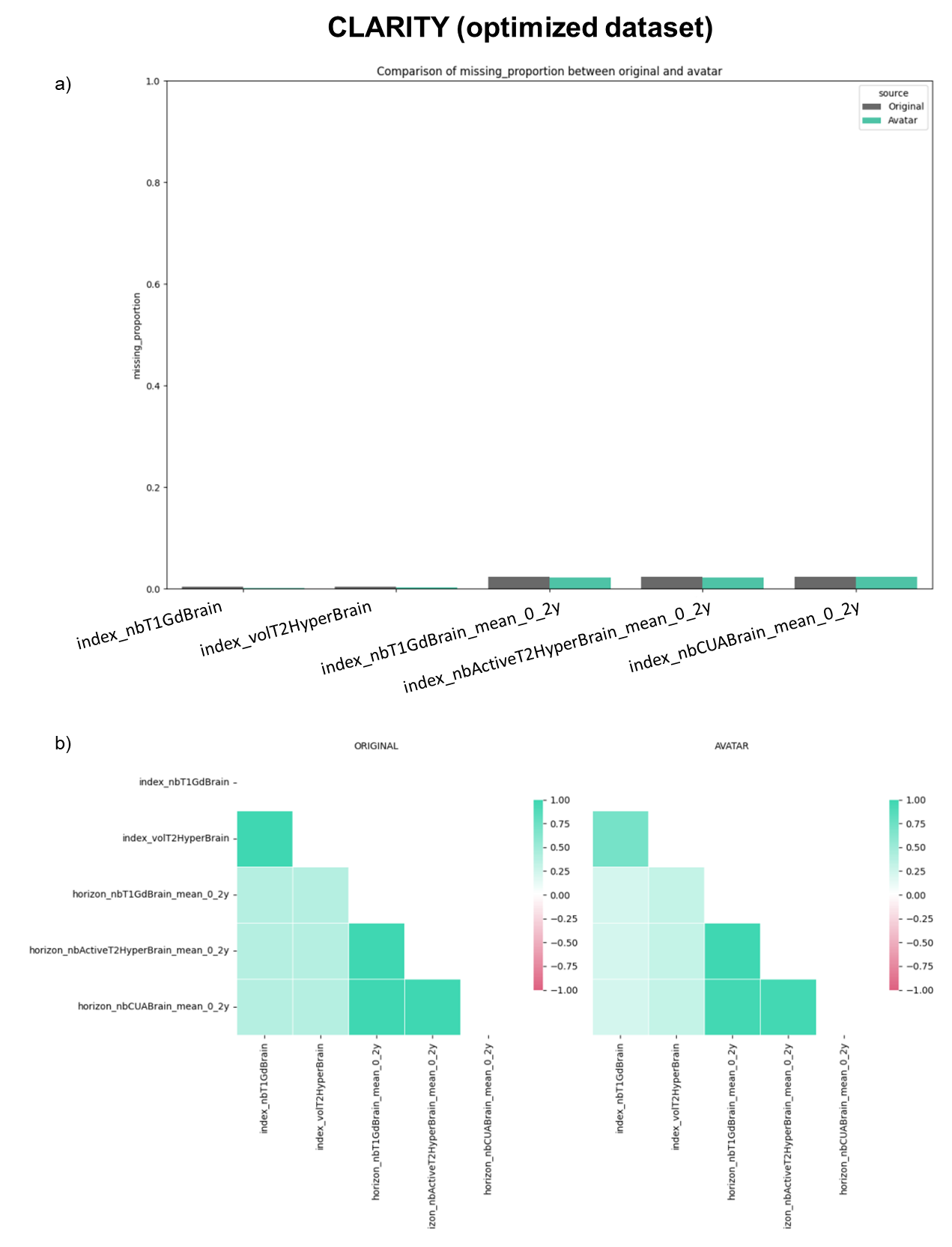 |
| --- |
| **Figure S3.** Comparison of the missing rates. (A) The bivariate patterns of missingness and (b) the figures are extracted from the avatarization report generated by the avatars client. The encoding of the optimized ADVANCE dataset left no missing data, as missing categorical variables were encoded as “missing” and missing quantitative data were encoded as aberrant negative values. |

Replication of the flowchart and tables of CLARITY

According to the RCT publication (https://doi.org/10.1056/nejmoa0902533).

| 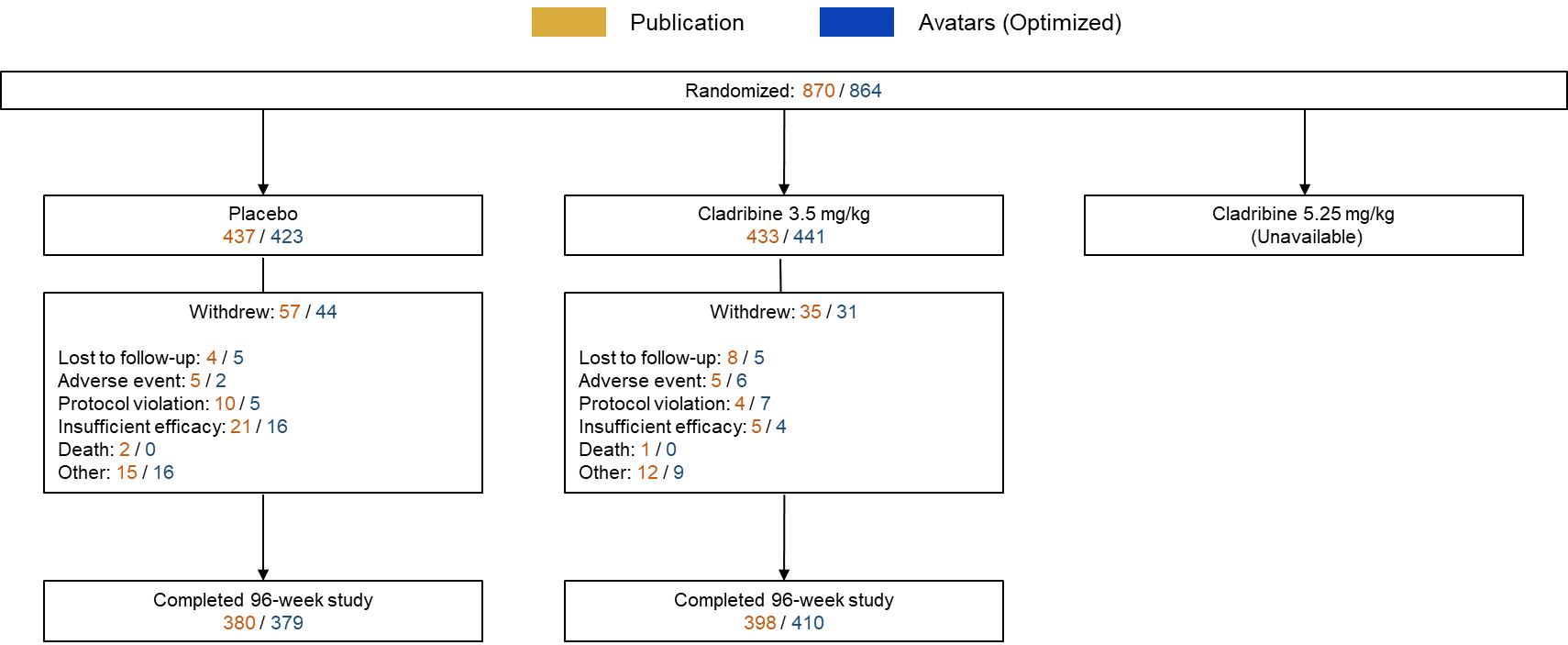 |
| --- |
| **Figure S4.** Replication of the CLARITY flow chart. It is supplementary Figure 2 in the RCT report: “Patient Enrollment and Disposition.” Data from the Cladribine 5.25 mg/kg arm were not available. |

| \| Variable \| Placebo  (N = 437 / 423) \| Cladribine 3.5 mg/kg  (N = 433 / 441) \| \| --- \| --- \| --- \| \| Age — yr \|  \|  \| \| Mean \| 38.7±9.9  38.1±7.0 \| 37.9±10.3  39.8±7.9 \| \| Range \| 18–64  20-58 \| 18–65  21-59 \| \| Female sex — no. (%) \| 288 (65.9)  278 (65.7) \| 298 (68.8)  317 (71.9) \| \| Mean weight — kg \| 70.3±15.4  69.5±10.7 \| 68.1±14.6  69.5±10.4 \| \| Disease duration from first onset — yr \|  \|  \| \| Mean \| 8.9±7.4  8.1±4.6 \| 7.9±7.2  8.2±5.6 \| \| Range \| 0.4–39.5  0.6-30.4 \| 0.3–42.3  1.0-34.6 \| \| EDSS score \|  \|  \| \| 0 — no. (%) \| 13 (3.0)  5 (1.2) \| 12 (2.8)  2 (0.5) \| \| 1 — no. (%) \| 70 (16.0)  22 (5.2) \| 75 (17.3)  40 (9.1) \| \| 2 — no. (%) \| 127 (29.1)  150 (35.5) \| 133 (30.7)  142 (32.2) \| \| 3 — no. (%) \| 96 (22.0)  152 (35.9) \| 108 (24.9)  157 (35.6) \| \| 4 — no. (%) \| 83 (19.0)  67 (15.8) \| 71 (16.4)  74 (16.8) \| \| ≥5 — no. (%) \| 48 (11.0)  27 (6.4) \| 34 (7.9)  26 (5.9) \| \| Mean score \| 2.9±1.3  2.8±1.0 \| 2.8±1.2  2.8±1.0 \| \| Gadolinium-enhancing T1-weighted lesions \|  \|  \| \| Patients with lesions — no. (%) \| 128 (29.3)  190 (44.9) \| 138 (31.9)  171 (38.9) \| \| Mean no. of lesions \| 0.8±2.1  1.0±1.9 \| 1.0±2.7  0.6±1.3 \| \| Mean volume of T2-weighted lesions — mm3 \| 14,287.6±13,104.8  14,458±9,899 \| 14,828.0±16,266.8  12,078±8,785 \| |
| --- | --- | --- | --- | --- | --- | --- | --- | --- | --- | --- | --- | --- | --- | --- | --- | --- | --- | --- | --- | --- | --- | --- | --- | --- | --- | --- | --- | --- | --- | --- | --- | --- | --- | --- | --- | --- | --- | --- | --- | --- | --- | --- | --- | --- | --- | --- | --- | --- | --- | --- | --- | --- | --- | --- | --- | --- | --- | --- | --- | --- | --- | --- | --- |
| **Table S3.** Replication of the CLARITY baseline characteristics table. It is Table 1 in the RCT report: “Demographic and Clinical Characteristics of the Patients at Baseline (Intention-to-Treat Population)”. The publication and avatar results are respectively in brown and blue. Plus–minus values are means ±SD. Percentages may not total 100 because of rounding. |

| \| End point \| Placebo  (N = 437 / 423) \| Cladribine 3.5 mg/kg  (N = 433 / 441) \| \| --- \| --- \| --- \| \| Relapse rate (primary end point) \|  \|  \| \| Annualized relapse rate (95% CI) \| 0.33 (0.29–0.38)  0.33 (0.29–0.38) \| 0.14 (0.12–0.17)  0.12 (0.10-0.15) \| \| Relative reduction in annualized relapse rate for cladribine vs. placebo — % \|  \| 57.6  62.3 \| \| P value \|  \| <0.001  <0.001 \| \| Relapse-free rate \|  \|  \| \| Patients without relapse — no. (%) \| 266 (60.9)  250 (59.1) \| 345 (79.7)  367 (83.2) \| \| Odds ratio for cladribine vs. placebo (95% CI) \|  \| 2.53 (1.87–3.43)  3.43 (2.50-4.73) \| \| P value \|  \| <0.001  <0.001 \| \| Relapse at 96 weeks \|  \|  \| \| No. of relapses — no. of patients (%) \|  \|  \| \| 0 \| 266 (60.9)  250 (59.1) \| 345 (79.7)  367 (83.2) \| \| 1 \| 109 (24.9)  115 (27.2) \| 69 (15.9)  56 (12.7) \| \| 2 \| 44 (10.1)  41 (9.7) \| 13 (3.0)  13 (2.9) \| \| 3 \| 15 (3.4)  17 (4.0) \| 5 (1.2)  5 (1.1) \| \| ≥4 \| 3 (0.7)  0 (0.0) \| 1 (0.2)  0 (0.0) \| \| Need for rescue therapy \|  \|  \| \| Patients receiving rescue therapy — no. (%) \| 27 (6.2)  28 (6.6) \| 11 (2.5)  10 (2.3) \| \| Odds ratio for cladribine vs. placebo (95% CI) \|  \| 0.40 (0.19–0.81)  0.34 (0.16-0.70) \| \| P value \|  \| 0.01  0.0047 \| \| Time to first relapse \|  \|  \| \| Hazard ratio for cladribine vs. placebo (95% CI) \|  \| 0.44 (0.34–0.58)  0.50 (0.32-0.77) \| \| P value \|  \| <0.001  0.002 \| \| Time to 3-mo sustained change in EDSS score \|  \|  \| \| Hazard ratio for cladribine vs. placebo (95% CI) \|  \| 0.67 (0.48–0.93)  0.54 (0.39-0.75) \| \| P value \|  \| 0.02  <0.001 \| \| Patients without a 3-mo sustained change in EDSS score \|  \|  \| \| Patients with no change — no. (%) \| 347 (79.4)  325 (76.8) \| 371 (85.7)  384 (87.0) \| \| Odds ratio for cladribine vs. placebo (95% CI) \|  \| 1.55 (1.09–2.22)  1.95 (1.35-2.81) \| \| P value \|  \| 0.02  0.01 \| \| Lesion activity on brain MRI \|  \|  \| \| Gadolinium-enhancing T1-weighted lesions \|  \|  \| \| Mean no. \| 0.91  0.77 \| 0.12  0.13 \| \| Relative reduction — % \|  \| 85.7  83.0 \| \| Active T2-weighted lesions \|  \|  \| \| Mean no. \| 1.43  1.28 \| 0.38  0.39 \| \| Relative reduction — % \|  \| 73.4  70.0 \| \| Combined unique lesions \|  \|  \| \| Mean no. \| 1.72  1.50 \| 0.43  0.43 \| \| Relative reduction — % \|  \| 74.4  71.0 \| |
| --- | --- | --- | --- | --- | --- | --- | --- | --- | --- | --- | --- | --- | --- | --- | --- | --- | --- | --- | --- | --- | --- | --- | --- | --- | --- | --- | --- | --- | --- | --- | --- | --- | --- | --- | --- | --- | --- | --- | --- | --- | --- | --- | --- | --- | --- | --- | --- | --- | --- | --- | --- | --- | --- | --- | --- | --- | --- | --- | --- | --- | --- | --- | --- | --- | --- | --- | --- | --- | --- | --- | --- | --- | --- | --- | --- | --- | --- | --- | --- | --- | --- | --- | --- | --- | --- | --- | --- | --- | --- | --- | --- | --- | --- | --- | --- | --- | --- | --- | --- | --- | --- | --- | --- | --- | --- | --- | --- | --- | --- | --- | --- | --- | --- | --- | --- | --- | --- | --- | --- | --- |
| **Table S4.** Replication of the endpoints table of CLARITY. It is Table 2 in the RCT report: “Clinical and Imaging End Points and Relapses during the 96-week Study (Intention-to-Treat Population)”. The publication and avatar results are respectively in brown and blue. |

| \| Adverse Event \| Placebo  (N = 435 / 423) \| Cladribine 3.5 mg/kg  (N = 430 / 441) \| \| --- \| --- \| --- \| \| Any adverse event — no. of patients (%) \| 319 (73.3)  312 (73.8) \| 347 (80.7)  353 (80.1) \| \| Most common adverse events — no. of patients (%) \|  \|  \| \| Headache \| 75 (17.2)  72 (17.0) \| 104 (24.2)  106 (24.0) \| \| Lymphocytopenia \| 8 (1.8)  20 (4.7) \| 93 (21.6)  86 (19.5) \| \| Nasopharyngitis \| 56 (12.9)  50 (11.8) \| 62 (14.4)  65 (14.7) \| \| Upper respiratory tract infection \| 42 (9.7)  48 (11.3) \| 54 (12.6)  48 (10.9) \| \| Nausea \| 39 (9.0)  32 (7.6) \| 43 (10.0)  45 (10.2) \| \| Any serious adverse event — no. of patients (%) \| 28 (6.4)  17 (4.0) \| 36 (8.4)  29 (6.6) \| \| Infections and infestations \| 7 (1.6)  3 (0.7) \| 10 (2.3)  5 (1.1) \| \| Neoplasms (benign, malignant, and unspecified) \| 0  0 \| 6 (1.4)  5 (1.1) \| \| Death \| 2 (0.5)  2 (0.5) \| 2 (0.5)  3 (0.7) \| |
| --- | --- | --- | --- | --- | --- | --- | --- | --- | --- | --- | --- | --- | --- | --- | --- | --- | --- | --- | --- | --- | --- | --- | --- | --- | --- | --- | --- | --- | --- | --- | --- | --- | --- | --- | --- | --- |
| **Supplementary Table 5. Replication of the endpoints table of CLARITY.** It is Table 3 in the RCT report: “Adverse Events and Investigator-Assessed Severity at 96 Weeks (Safety Population)”.  The publication and avatar results are respectively in brown and blue. The mild:severe event ratios could not be replicated due to the encoding of the adverse event variables as Booleans in the optimized dataset. |

**Figure S5.** Replication of the 2-year ADVANCE flowchart. It is Figure 1 in the 2-year RCT report: “Patient disposition – over 2 years”. N, n: number of subjects.

Replication of the flowchart and tables of ADVANCE

According to the 1-year and 2-year publications (<https://doi.org/10.1016/S1474-4422(14)70068-7> and <https://doi.org/10.1177/1352458514557986>). Adverse events and individual relapse data were not available.

| 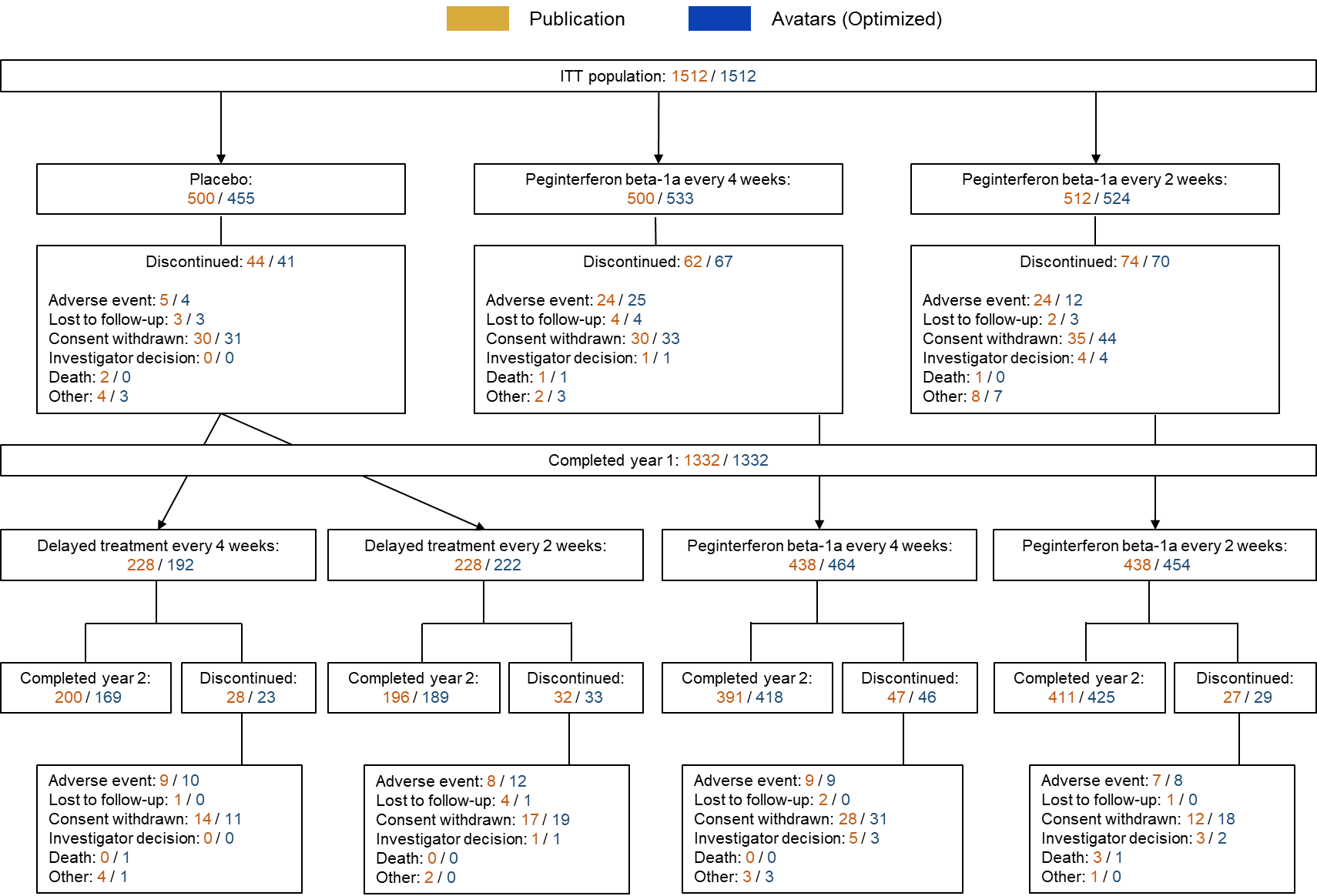 |
| --- |
|  |

**Table S6.** Replication of the baseline characteristics table of ADVANCE. It is Table 1 in the 1-year RCT report: “Baseline characteristics”. Data are mean (SD) or n (%).

| \|  \| Placebo group (n=500 / 455) \| Peginterferon beta-1a 125 μg every 2 weeks group  (n=512 / 524) \| Peginterferon beta-1a 125 μg every 4 weeks group  (n=500 / 533) \| \| --- \| --- \| --- \| --- \| \| Age (years) \| 36·3 (9·7)  35.9 (8.6) \| 36·9 (9·8)  37.1 (8.9) \| 36·4 (9·9)  36.3 (8.8) \| \| Women \| 358 (72%)  335 (74%) \| 361 (71%)  374 (71%) \| 352 (70%)  367 (69%) \| \| Time since first multiple sclerosis symptoms (years) \| 6·3 (6·3)  6.1 (5.7) \| 6·9 (6·6)  6.7 (6.0) \| 6·5 (6·1)  6.6 (5.6) \| \| Time since multiple sclerosis diagnosis (years) \| 3·5 (4·6)  3.3 (3.9) \| 4·0 (5·1)  3.9 (4.7) \| 3·4 (4·4)  3.4 (4.1) \| \| Relapses within the previous 3 years \| 2·6 (1·00)  2.5 (0.88) \| 2·6 (0·99)  2.5 (0.87) \| 2·5 (0·77)  2.4 (0.71) \| \| Relapses within the previous 12 months \| 1·6 (0·67)  1.5 (0.62) \| 1·6 (0·67)  1.6 (0.65) \| 1·5 (0·62)  1.5 (0.59) \| \| EDSS score \| 2·44 (1·18)  2.43 (1.06) \| 2·47 (1.26)  2.49 (1.20) \| 2·48 (1·24)  2.47 (1.09) \| \| <4 \| 432 (86%)  400 (88%) \| 423 (83%)  440 (84%) \| 413 (83%)  469 (88%) \| \| ≥4 \| 68 (14%)  55 (12%) \| 89 (17%)  84 (16%) \| 87 (17%)  64 (12%) \| \| Patients without gadolinium-enhancing lesions \| 59%  60% \| 65%  64% \| 59%  54% \| \| Number of gadolinium-enhancing lesions \| 1·6 (3·8)  1.4 (3.7) \| 1·2 (3·4)  1.1 (2.7) \| 1·8 (5·4)  2.0 (4.5) \| \| Number of T2 lesions \| 50·6 (35·7)  49.7 (33.5) \| 48·7 (36·8)  46.0 (31.4) \| 51·4 (36·0)  52.0 (31.8) \| |
| --- | --- | --- | --- | --- | --- | --- | --- | --- | --- | --- | --- | --- | --- | --- | --- | --- | --- | --- | --- | --- | --- | --- | --- | --- | --- | --- | --- | --- | --- | --- | --- | --- | --- | --- | --- | --- | --- | --- | --- | --- | --- | --- | --- | --- | --- | --- | --- | --- | --- | --- | --- | --- |
|  |

**Table S7.** Replication of the end points table of ADVANCE. It is Table 1 in the 2-year RCT report: “Summary of clinical and MRI endpoints over 2 years by original randomization group.” The hazard ratio of relapses could not be replicated because individual relapse event data were not available.

| \| End point \| Delayed treatment (n=500 / 455) \| Peginterferon beta-1a every 2 weeks  (n=512 / 524) \| Peginterferon beta 1a every 4 weeks  (n=500 / 533) \| \| --- \| --- \| --- \| --- \| \| Annualized relapse rate at 2 years \|  \|  \|  \| \| Annualized relapse rate (95% CI) \| 0.351 (0.295, 0.418)  0.32 (0.27, 0.36) \| 0.221 (0.183, 0.267)  0.21 (0.17, 0.25) \| 0.291 (0.244, 0.348)  0.25 (0.22, 0.29) \| \| Rate ratio vs. delayed treatment (95% CI) \|  \| 0.629 (0.500, 0.790)  0.63 (0.50, 0.79) \| 0.829 (0.666, 1.030)  0.82 (0.66, 1.02) \| \| p-value vs. delayed treatment \|  \| <0.0001  <0.0001 \| 0.0906  0.072 \| \| Rate ratio every 2 weeks vs. every 4 weeks (95% CI) \|  \| 0.759 (0.600, 0.959)  0.77 (0.61, 0.96) \|  \| \| p-value (every 2 weeks vs. every 4 weeks) \|  \| 0.0209  0.023 \|  \| \| Estimated proportion of patients with a relapse at 2 year \|  \|  \|  \| \| Number of patients relapsed \| 192  160 \| 124  122 \| 158  158 \| \| Proportion relapsed \| 0.402  0.352 \| 0.265  0.233 \| 0.344  0.296 \| \| Disability progression at 2 years (12-week confirmed) \|  \|  \|  \| \| Number of patients with disability progression \| 75  59 \| 51  37 \| 56  64 \| \| Estimated proportion with disability progression \| 0.162  0.132 \| 0.112  0.08 \| 0.123  0.135 \| \| Hazard ratio vs. delayed treatment (95% CI) \|  \| 0.67 (0.47, 0.95)  0.54 (0.36, 0.82) \| 0.75 (0.53, 1.05)  0.96 (0.68, 1.37) \| \| p-value vs. delayed treatment \|  \| 0.0257  0.003 \| 0.0960  0.83 \| \| Hazard ratio every 2 weeks vs. every 4 weeks (95% CI) \|  \| 0.89 (0.61, 1.31)  0.56 (0.37, 0, 84) \|  \| \| p-value (every 2 weeks vs. every 4 weeks) \|  \| 0.5665  0.005 \|  \| \| Disability progression at 2 years (24-week confirmed) \|  \|  \|  \| \| Number of patients with disability progression \| 57  54 \| 34  28 \| 52  57 \| \| Estimated proportion with disability progression \| 0.119  0.102 \| 0.077  0.06 \| 0.113  0.121 \| \| Hazard ratio vs. delayed treatment (95% CI) \|  \| 0.59 (0.38, 0.90)  0.56 (0.35, 0.91) \| 0.91 (0.63, 1.33)  1.16 (0.78, 1.73) \| \| p-value vs. delayed treatment \|  \| 0.0137  0.019 \| 0.6243  0.46 \| \| Hazard ratio every 2 weeks vs. every 4 weeks (95% CI) \|  \| 0.64 (0.42, 0.99)  0.49 (0.31, 0.76) \|  \| \| p-value (every 2 weeks vs. every 4 weeks) \|  \| 0.0459  0.002 \|  \| \| New or newly enlarging T2-weighted hyperintense lesions at 2 years \|  \|  \|  \| \| Number of patients evaluated \| 393  414 \| 407  456 \| 389  465 \| \| Adjusted mean number of lesions \| 14.8  16.5 \| 5.0  5.6 \| 12.5  15.6 \| \| Lesion mean ratio (peginterferon beta-1a:delayed treatment) (95% CI) \|  \| 0.33 (0.27, 0.41)  0.38 (0.32, 0.46) \| 0.84 (0.69. 1.03)  0.95 (0.79, 1.14) \| \| p-value (peginterferon beta-1a: delayed treatment) \|  \| <0.0001  <0.0001 \| 0.0973  0.59 \| \| Lesion mean ratio (every 2 weeks: every 4 weeks) (95% CI) \|  \| 0.40 (0.32, 0.49)  0.40 (0.34, 0.48) \|  \| \| *P* value (every 2 weeks vs. every 4 weeks) \|  \| <0.0001  <0.0001 \|  \| \| Gd+ lesions at 2 years \|  \|  \|  \| \| Number of patients evaluated \| 393  414 \| 407  456 \| 389  465 \| \| Mean number of lesions (SE) \| 0.5 (0.08)  0.2 \| 0.2 (0.06)  0.4 \| 0.7 (0.12)  0.7 \| \| p-value (peginterferon beta-1a vs delayed treatment) \|  \| 0.0002  <0.0001 \| 0.2169  0.16 \| \| Percent reduction (every 2 weeks vs. every 4 weeks) \|  \| 71  141 (increase) \|  \| \| p-value (every 2 weeks vs. every 4 weeks) \|  \| <0.0001  <0.0001 \|  \| |
| --- | --- | --- | --- | --- | --- | --- | --- | --- | --- | --- | --- | --- | --- | --- | --- | --- | --- | --- | --- | --- | --- | --- | --- | --- | --- | --- | --- | --- | --- | --- | --- | --- | --- | --- | --- | --- | --- | --- | --- | --- | --- | --- | --- | --- | --- | --- | --- | --- | --- | --- | --- | --- | --- | --- | --- | --- | --- | --- | --- | --- | --- | --- | --- | --- | --- | --- | --- | --- | --- | --- | --- | --- | --- | --- | --- | --- | --- | --- | --- | --- | --- | --- | --- | --- | --- | --- | --- | --- | --- | --- | --- | --- | --- | --- | --- | --- | --- | --- | --- | --- | --- | --- | --- | --- | --- | --- | --- | --- | --- | --- | --- | --- | --- | --- | --- | --- | --- | --- | --- | --- | --- | --- | --- | --- | --- | --- | --- | --- | --- | --- | --- | --- | --- | --- | --- | --- | --- | --- | --- | --- | --- | --- | --- | --- | --- | --- | --- | --- |
|  |
